# From Inside Out: How the Buried Interface, Shell Defects, and Surface Chemistry Conspire to Determine Optical Performance in Nonblinking Giant Quantum Dots

**DOI:** 10.1002/smsc.202300092

**Published:** 2023-10-10

**Authors:** Ajay Singh, Somak Majumder, Noah J. Thompson Orfield, Ibrahim Sarpkaya, Dennis Nordlund, Karen C. Bustillo, Jim Ciston, Victoria Nisoli, Sergei A. Ivanov, Eric G. Bowes, Han Htoon, Jennifer A. Hollingsworth

**Affiliations:** ^1^ Materials Physics & Applications Division Center for Integrated Nanotechnologies Los Alamos National Laboratory Los Alamos NM 87545 USA; ^2^ Stanford Synchrotron Radiation Light Source SLAC National Accelerator Laboratory Stanford CA 94309 USA; ^3^ National Center for Electron Microscopy, Molecular Foundry Lawrence Berkeley National Laboratory 1 Cyclotron Road Berkeley CA 94720 USA; ^4^ Present address: Bilkent University UNAM – National Nanotechnology Research Center Ankara 06800 Turkey

**Keywords:** giant quantum dots, interfacial alloying, materials-by-design, structure–function correlations

## Abstract

“Giant” or core/thick‐shell quantum dots (gQDs) are an important class of solid‐state quantum emitter characterized by strongly suppressed blinking and photobleaching under ambient conditions, and reduced nonradiative Auger processes. Together, these qualities provide distinguishing and useful functionality as single‐ and ensemble‐photon sources. For many applications, operation at elevated temperatures and under intense photon flux is desired, but performance is strongly dependent on the synthetic method employed for thick‐shell growth. Here, a comprehensive analysis of gQD structural properties “from the inside out” as a function of shell‐growth method is reported: successive ionic layer adsorption and reaction (SILAR) and high‐temperature continuous injection (HT‐CI), or sequential combinations of the two. Key correlations across synthesis methods, structural features (interfacial alloying, stacking‐fault density and surface‐ligand identity), and performance metrics (quantum yield, single‐gQD photoluminescence under thermal/photo stress, charging behavior and quantum‐optical properties) are identified. Surprisingly, it is found that interfacial alloying is the strongest indicator of gQD stability under stress, but this parameter is not the determining factor for Auger suppression. Furthermore, quantum yield is strongly influenced by surface chemistry and can approach unity even in the case of high shell‐defect density, while introduction of zinc‐blende stacking faults increases the likelihood that a gQD exhibits charged‐state emission.

## Graphical abstract

The functionally unique “giant” quantum dot—a nonblinking and nonphotobleaching room‐temperature photon source—is the subject of numerous investigations of its optical properties and application demonstrations from 3D single‐molecule tracking to light‐emitting diodes. In this work, explicit synthesis–structure–function correlations are revealed as a blueprint for designing syntheses to produce nanoscale structures for long‐term stability under harsh operating conditions of high‐temperature and photon flux.

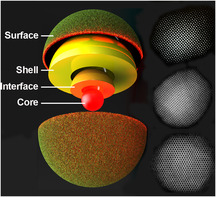

## 1 Introduction

A class of colloidal quantum dot (QD), known as the giant QD (gQD), is synthetically engineered to suppress nonradiative processes that are characteristic of other QDs. Structurally, gQDs are a type of core/shell QD in which the shell is particularly thick (typically (>10 monolayers (MLs)). Functionally, gQDs exhibit strongly suppressed or nonexistent fluorescence intermittency (blinking between “on” and “off” states) and resistance to permanent photobleaching.^[^ ^1^, ^2^, ^3^ ^]^ The extent to which these properties manifest can depend on both shell thickness and core size.^[^ ^4^ ^]^ In this way, several reports have attempted to quantify the onset of nonblinking behavior using a particle volume threshold, but direct comparisons are complicated by the use of different experimental parameters for the blinking analyses, such as distinct excitation wavelength, pump fluence, or observation time.^[^ ^4^, ^5^, ^6^ ^]^ Nevertheless, clear gQD properties of suppressed blinking and photobleaching or extreme photostability have now been reported for II–VI, III–V, I–III–VI, and IV–VI QD‐core compositions, including alloy compositions, for emission from the blue‐visible to the infrared,^[^ ^1^, ^2^, ^3^, ^4^, ^5^, ^6^, ^7^, ^8^, ^9^, ^10^, ^11^, ^12^, ^13^, ^14^ ^]^ as well as novel configurations that afford two‐color blinking‐suppressed emission^[^ ^15^, ^16^ ^]^ or hybrid photonic–plasmonic functionality.^[^ ^17^ ^]^

The core/thick‐shell structure of the gQD enables superior performance in blinking and photobleaching by fundamentally altering underlying photophysical properties compared to conventional core/shell QDs. Band‐edge or hot carrier trapping and Auger‐mediated recombination of charged states (e.g., an exciton plus an extra carrier, or trion) have been identified as processes that lead to blinking.^[^ ^18^, ^19^ ^]^ These processes also contribute to irreversible photobleaching observed under conditions of high photon flux and elevated temperature.^[^ ^20^ ^]^ The effect of a “giant” shell on carrier trapping is fairly straightforward; it serves to more effectively separate excited‐state carriers in the core from surface trap states compared to a thin shell.^[^ ^18^ ^]^ The factors underlying suppression of Auger recombination are less obvious, but the impact is clear—when Auger is suppressed, charged states and multiple‐exciton states can recombine radiatively with high efficiency, i.e., they are not “dark.”^[^ ^21^, ^22^, ^23^, ^24^, ^25^, ^26^, ^27^, ^28^ ^]^

Several structural factors contribute to Auger suppression in gQDs. First, the larger volume of these nanocrystals compared to conventional QDs reduces carrier–carrier/exciton–exciton coupling and, thereby, contributes to a reduced Auger effect,^[^ ^29^ ^]^ where even use of a large core (e.g., ≈5 nm in the case of CdSe) contributes to the Auger‐rate‐reducing weakened confinement.^[^ ^24^, ^26^ ^]^ However, it was reported that the impact of increased particle volume is not sufficient to explain the strong Auger suppression of some gQDs.^[^ ^22^ ^]^ A quasi type II or type II band alignment causes partial or complete spatial separation of electron–hole carriers between the core and the shell regions of the QD, respectively. In this case, lower Auger efficiencies result from the reduced electron–hole overlap and repulsive Coulombic interactions that can also develop.^[^ ^30^, ^31^ ^]^ Indeed, the gQD compositions for which evidence for Auger suppression is most clear, CdSe/CdS and InP/CdS QDs, are characterized by quasi type II and type II band structures, respectively.^[^ ^8^, ^21^, ^22^, ^23^ ^]^ In contrast, even basic blinking suppression is not accessible in the case of type I CdSe/ZnS QDs with increasing shell thickness.^[^ ^32^ ^]^ In addition to particle volume and band structure, a structural feature frequently used to explain strongly suppressed Auger in gQDs is compositional alloying at the core/shell interface. An alloyed interface would smooth the confinement potential between core and shell and, if present, could have a strong impact on Auger.^[^ ^33^ ^]^ Thus, both carrier‐trapping and Auger processes can have important and nonequal impacts on blinking behavior and long‐term photostability in QDs. One pathway can dominate over the other, and the extent to which this happens is both system and shell‐thickness dependent.^[^ ^19^, ^20^, ^34^ ^]^

**Figure 1:**
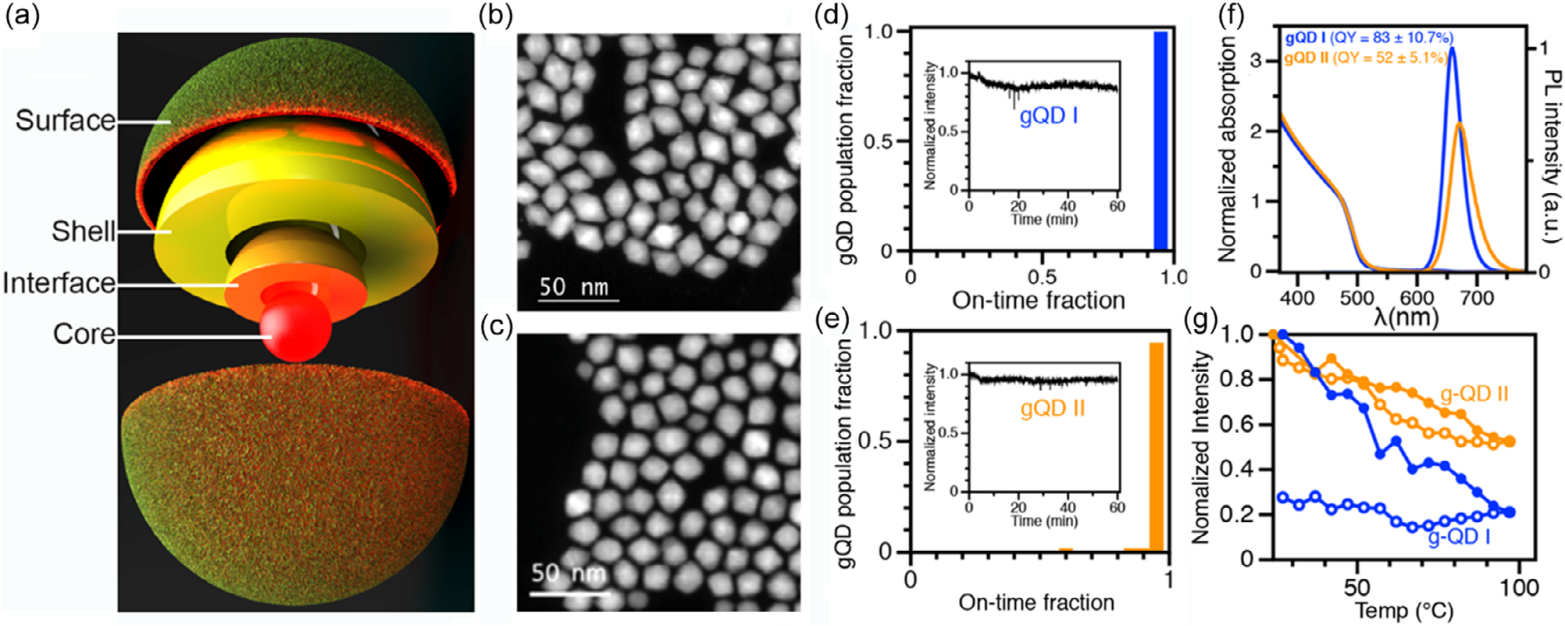
Attributes of the gQD subjects under investigation. a) Schematic illustration of the components of the core/shell QD from the inside out. b,c) HAADF‐STEM images of gQD I (b) and gQD II (c) nanocrystals. d,e) gQD population fraction as a function of on‐time fraction for gQD I (54 QDs analyzed) (d) and gQD II (64 QDs analyzed) (e) under high pump‐fluence widefield excitation and collection conditions (1 W mm^−2^, 405 nm continuous‐wave excitation, room temperature). The insets show minimal photobleaching in each case over 1 h. f) Absorption (onset ≈515 nm) and absorption‐normalized emission spectra for gQD I (blue) and gQD II (orange). g) Photoluminescence intensity as a function of temperature for gQD I (blue) and gQD II (orange) under ultrahigh photon flux (15 W mm^−2^) revealing differences in extent of photobleaching and recovery upon cooling (≈2.5 h of illumination under heating/cooling stress).

Although elements of the “design criteria” for controlling the processes that influence fluorescence stability over short (blinking) and long (bleaching) timescales are roughly known, as described above, their relative importance and the explicit correlations between structural parameter and functional parameter remain to be established. Here, toward enabling the goal of precision nanoscale synthesis and functionality‐by‐synthetic design, we undertake a thorough assessment of synthesis–structure–property relationships in the prototype gQD system—CdSe/CdS. We employ as test subjects CdSe/CdS gQDs that have been prepared using different synthetic methods. In both cases, the gQDs comprise the same CdSe core, but the CdS shell‐growth method is different–modified successive ionic layer adsorption and reaction (SILAR)^[^ ^4^ ^]^ versus high‐temperature continuous injection (HT‐CI).^[^ ^5^, ^20^ ^]^ We have previously shown that the choice of shell‐growth method has a profound impact on gQD functionality, especially quantum yield (QY) and long‐term photostability.^[^ ^20^ ^]^ In this study, we correlate these performance anomalies with specific features of structure and composition using both single‐QD and ensemble methods to characterize the nanocrystals from the inside out, i.e., from the core/shell interface to the QD surface (**Figure** **1**a). For the first time, we quantify the extent of interfacial compositional mixing (alloying) using direct methods; we determine the number and origin of shell defects, and we characterize the chemical nature of the QD surface. Each parameter is correlated with both the causal synthesis parameter and ensuing differences in gQD performance, allowing unprecedented insight for the rational design of new functional nanomaterials from synthesis to structure and performance. Surprisingly, we find that increased interfacial alloying does not result in higher biexciton QYs, and surface chemistry has a stronger effect on exciton QY than shell defect density, while the latter may be beneficial for long‐term photostability due to a promotion of a photobleaching mechanism controlled by dimming rather than a catastrophic failure to emit photons.

## 2 Results

### 2.1 Two gQD Subjects

Two distinct CdSe/CdS gQDs were synthesized using the different shell‐growth procedures (HT‐CI and SILAR). The same CdSe core (≈5 nm) was used in each case, so that any differences observed in structural and photophysical properties arise during shell synthesis. Approximately 16 ML‐equivalents of CdS shell precursor were added in total, with aliquots extracted for intermediate shell thicknesses. The gQD prepared using the high‐temperature continuous injection method is referred to here as gQD I and that prepared using the SILAR method is gQD II (see Supporting Information for synthetic details). Low‐resolution scanning transmission electron microscopy (STEM) images reveal the typical morphology for each gQD (Figure 1b,c), which approximates a truncated octahedron. gQD I nanocrystals are relatively elongated compared to gQD II nanocrystals—gQD I: 20.7 ± 2.1 nm × 15.6 ± 1.4 nm, or aspect ratio of 1.33; gQD II: 19.1 ± 1.2 nm × 16.5 ± 1.3 nm, or aspect ratio of 1.16 (80 QDs were measured using ImageJ analysis in each case; see Figure S1a, Supporting Information). Given a CdS ML thickness of 0.3375 nm, the observed gQD particle widths constitute a shell thickness of ≈16–17 MLs.

gQD I and gQD II exhibit typical gQD behavior with respect to room‐temperature blinking and photobleaching, where nearly 100% of the QDs do not blink off over 1 hour of observation under continuous excitation (405 nm CW laser; 1 W mm^−2^). Furthermore, neither type of gQD photobleaches to a significant extent, with gQD I retaining ≈90% of its original intensity and gQD II retaining ≈95% intensity (Figure 1d,e). The gQDs are also similar with respect to ensemble optical absorption. The thick CdS shell dominates the spectrum, and the principal absorption onset occurs at ≈515 nm, or 2.4 eV, which is the bulk bandgap energy of CdS (Figure 1f).

Despite the similarities in room‐temperature single‐QD stabilities, differences become apparent when other properties are compared. First, the photoluminescence (PL) peak position for gQD II is redshifted compared to that for gQD I, and its full‐width‐at‐half‐maximum (FWHM) height is larger (44.6 nm compared to 34.1 nm, respectively). Furthermore, the QY for gQD II is significantly lower: gQD II = 52 ± 5.1% and gQD I = 83 ± 10.7% (Figure 1f). In contrast with this efficiency metric, however, gQD II outperforms when stability is studied outside ambient conditions. Namely, the gQDs were interrogated with a laser operating at a high photon flux of 15 W mm^−2^, and the temperature was cycled from room temperature to ≈100 °C and back to room temperature over ≈2.5 h. PL from single nanocrystals was monitored during both heat‐up and cool‐down (Figure 1g). Both gQDs exhibited thermal quenching, but gQD I nanocrystals lost more of their original intensity and, in contrast with gQD II behavior, the loss was irreversible. A similar trend was observed when heating was conducted under lower excitation power (1 W mm^−2^), for which the gQDs were subjected to multiple heating and cooling cycles. Over two cycles, gQD I exhibited progressively increasing thermal quenching and permanent (irreversible) photobleaching, while gQD II exhibited less relative change in QY and almost complete recovery following each thermal quench (Figure S1, Supporting Information).

Thus, despite nominally similar structures—identical CdSe core and comparable “giant” CdS shell—gQD I and gQD II are unalike in terms of PL efficiency and PL stability. These are key performance criteria for a range of real‐world QD applications from single‐molecule tracking^[^ ^35^, ^36^, ^37^ ^]^ to display technologies,^[^ ^38^, ^39^, ^40^ ^]^ solid‐state lighting,^[^ ^41^, ^42^, ^43^, ^44^, ^45^, ^46^, ^47^ ^]^ and single‐photon generation.^[^ ^48^, ^49^ ^]^ Notably, in the case of light‐emitting diode (LED) technologies, the thick shell has other benefits that are not affected by the choice of shell‐growth method. Namely, the shell acts as a spacer between emissive cores, which decreases the efficiency of QD‐to‐QD nonradiative energy transfer.^[^ ^41^, ^43^, ^45^, ^46^, ^47^ ^]^ Also, the size of the shell compared to the size of the core (e.g., commonly ≈95:5 by volume) means that absorption takes place primarily in the shell, while emission originates in the core, resulting in a large effective Stokes shift and minimal self‐reabsorption.^[^ ^1^, ^42^ ^]^ Due both to reduced energy transfer and self‐reabsorption, close‐packed solid‐state films can retain the QY of the solution phase.^[^ ^43^ ^]^ Nevertheless, the need for near‐unity QY coupled with long‐term stability under device‐relevant conditions remains a critical bottleneck. To understand the structural origin of synthesis‐dependent differences for this key gQD functionality, we now look “inside” the nanocrystals for otherwise hidden differences in composition and structure (Figure 1a).

### 2.2 Probing Compositional Abruptness at the Buried Interface

**Figure 2:**
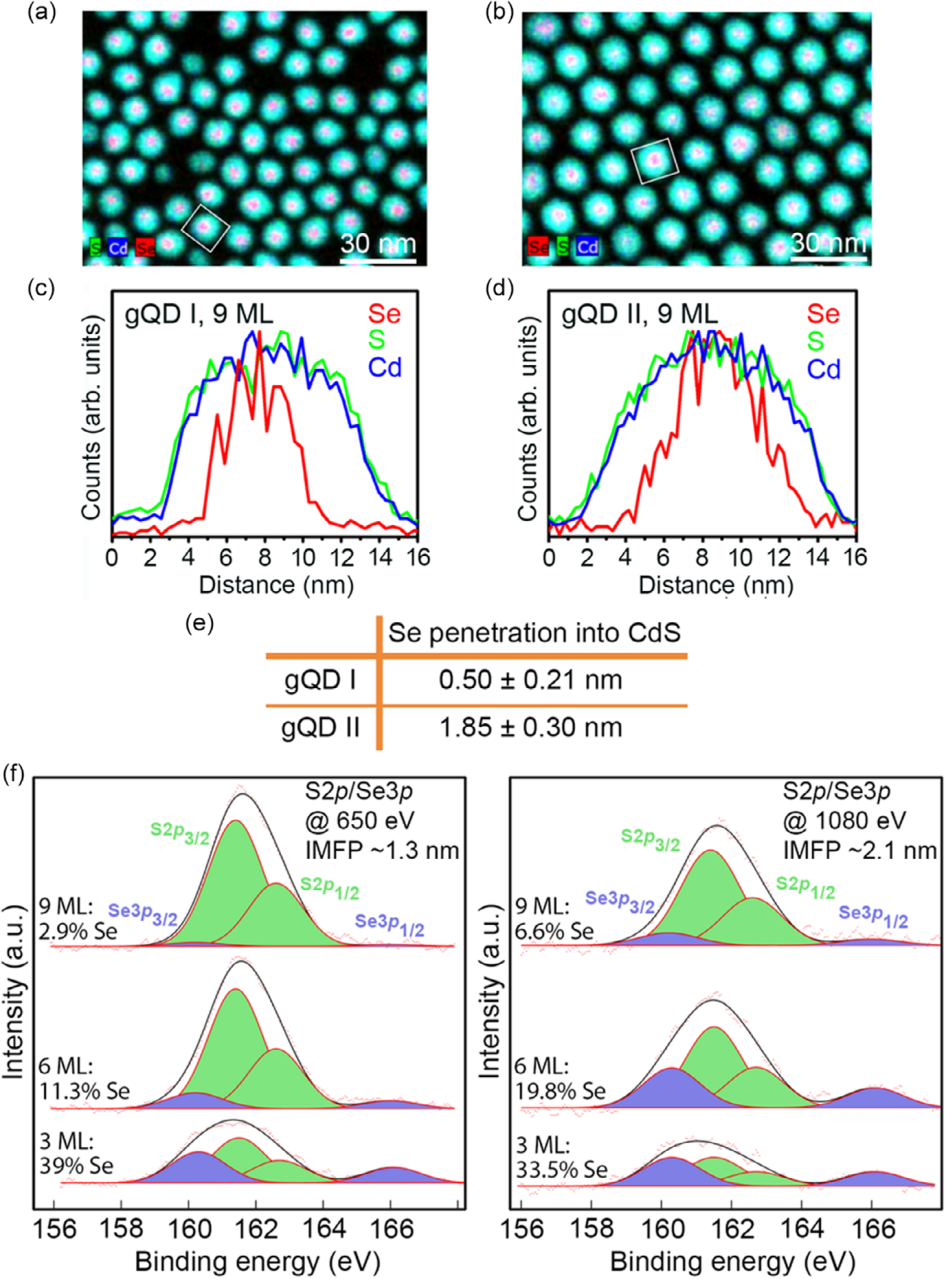
Assessing the buried core/shell interface and the extent of Se excursion from the CdSe core at the level of individual nanocrystals and for the ensemble. a,b) STEM–EDS images of core/shell nanocrystals possessing ≈9 MLs of CdS shell from gQD I (a) and gQD II (b) syntheses. c,d) EDS line scan analyses of the QDs indicated in (a) and (b) by squares. e) Average Se penetration into the CdS shell obtained for each gQD by analysis in each case of ≈30 nanocrystals. f) Ensemble technique—energy‐dependent XPS—corroborates extensive excursion of Se into the shell for gQD II via ensemble analysis: XPS spectra for CdSe/CdS core/shell QDs possessing three different shell thicknesses (3, 6, and 9 MLs) showing the doublet peaks for S 2p and Se 3p orbitals obtained using a photon energy of either 650 eV (left) or 1080 eV (right). IMFP is shown for each energy in the respective panels.

Using STEM–energy‐dispersive X‐ray spectroscopy (STEM–EDS) elemental mapping (see Supporting Information for experimental details), we investigate the nature of the core/shell interface and, in particular, whether it is abrupt or alloyed. In composite EDS maps (all elements represented by different colors are overlayed; Cd: blue, Se: red, S: green), the core/shell structure of both gQDs is revealed (**Figure** **2**a,b; Figure S2, Supporting Information, shows individual element EDS maps). Differences between gQD I and gQD II are apparent when the composite maps are analyzed by plotting the EDS counts for each element, as shown in Figure 2c,d for representative gQDs. It is observed that Se atoms diffuse from the core to the shell, suggesting that the interface is not compositionally abrupt, as quantified in Figure 2e.

The extent to which Se spreads from the core into the shell is clearly different for the two gQDs. We note that in order to access the buried core/shell interface by this method the shells used for analysis could not be fully “giant.” However, the shells needed to be sufficiently thick to afford adequate stability during the long EDS scan times required for acquisition of high‐quality datasets. To determine whether a given shell thickness was stable to the experiment, high‐angle annular dark‐field (HAADF)‐STEM images were acquired before and after EDS map acquisition and observed for signs of beam damage (see Supporting Information for details) The “goldilocks” shell thickness in this case was found to be 9 MLs. For 9 ML gQD I and II nanocrystals, Se signal was evident out to ≈6 nm and 8.5 nm, respectively, i.e., occupying a larger radial distance than the dimensions of the starting ≈5 nm CdSe core (see Table S1, Supporting Information data summary for multiple 9 ML QD batches).

It can be concluded that during shell growth some Se migrates into the shell, and this happens to a greater extent in the case of SILAR growth. In addition, the Se signal drops off abruptly at 0.50 ± 0.21 nm radially away from the core for gQD I nanocrystals, while it gradually decreases out to 1.85 ± 0.30 nm away from the core for gQD II nanocrystals (see steep vs shallower slope in red Se signal in Figure 2c compared to 2d, respectively). This finding indicates that the internal compositional structure for the two systems can be approximated as: CdSe/CdSeS/CdS (gQD I) and CdSe/CdSe_*x*_S_*y*(*y*<*x*)_/CdSe_*x*_S_*y*(*y*>*x*)_/CdS (gQD II), where the intermediate shell in the case of gQD II can be described as a graded alloy, rather than a single alloy composition. The analysis was performed on multiple nanocrystals (≈30 QDs per type) and also on different synthetic batches (duplicate syntheses; see additional maps and analysis in Supporting Information; Figure S2 and Table S1, Supporting Information).

Analysis by STEM–EDS employed multiple nanocrystals for a statistically relevant sampling. Nevertheless, relying solely on such single‐nanocrystal techniques can introduce bias if the selected sample is not adequately representative of the population. For this reason, we also investigated the core/shell interface using synchrotron‐based X‐ray photoelectron spectroscopy (XPS). Synchrotron XPS is an ensemble technique that allows elemental depth profiling due to variation in escape depth of photoemitted electrons from a sample exposed to different incident photon energies. Here, we investigated gQD II nanocrystals with increasing shell thicknesses from 3 to 6 to 9 MLs (Figure 2f). High‐resolution S 2p (green) and Se 3p (violet) core‐level spectra were obtained using two incident photon energies: 650 and 1080 eV, for which the respective inelastic mean free path lengths were calculated (Figure 2f insets; see Supporting Information for experimental details). Spectra collected for the 3 ML sample at both energies (Figure 2f, left: 650 eV; Figure 2f, right: 1080 eV) clearly show two distinct sets of spectral features corresponding to S 2p_3/2_–S 2p_1/2_ (green) and Se 3p_3/2_–Se 3p_1/2_ (violet) core levels. With increasing CdS shell thickness, the relative intensity of Se 3p compared to S 2p core level decreases. Though less visible in spectra obtained using 650 eV incident photon energy, the core Se signature is still clearly present in the 9 ML sample (≈3.3 nm shell thickness) when interrogated using the 1080 eV incident photon energy, which affords higher depth profiling (Figure 2f, right).

Considering the inelastic mean free path (IMFP) for each photon energy (Figure 2f insets) and the approximate thickness of a CdS ML (0.3375 nm), the photoelectrons at different kinetic energies have mean free path values that correspond to CdS shell thicknesses of ≈4 ML (650 eV photon energy; electron kinetic energy 390 eV) and ≈6 ML (1080 eV). In a first approximation, for Se to have a significant fraction in XPS spectra in each case, Se would have to extend from the core out to ≈5 and ≈3 ML of the shell, respectively (i.e., the difference between the penetration depth and the total shell thickness of ≈9 ML). More conservatively, taking into account the standard exponential decay for the outgoing electrons (IMFP is not a hard cutoff) and using a simple geometric model, we can estimate the expected anionic fractions for the different shell thicknesses. We find that only if we increase the “effective” core radius by about 1 nm can we reproduce the higher anionic Se fractions observed in the spectra, confirming that Se atoms are diffusing into the shell material. Significantly, the peak positions and peak splitting remain the same for all shell thicknesses and photon energies, which confirms that these peaks indeed represent the constituent elements of the nanocrystals. Thus, results from EDS and synchrotron XPS are consistent. In either case–the deep penetration of Se into the SILAR‐grown shell or the lesser escape observed for gQD I–the observed alloying is unintentional, which highlights the importance of directly characterizing this buried interface when attempting to draw conclusions about the possible impact of interfacial alloying on photophysical properties.

### 2.3 Quantifying Defects in the Shell and Assessing Their Origin at the Core/Shell Interface

**Figure 3:**
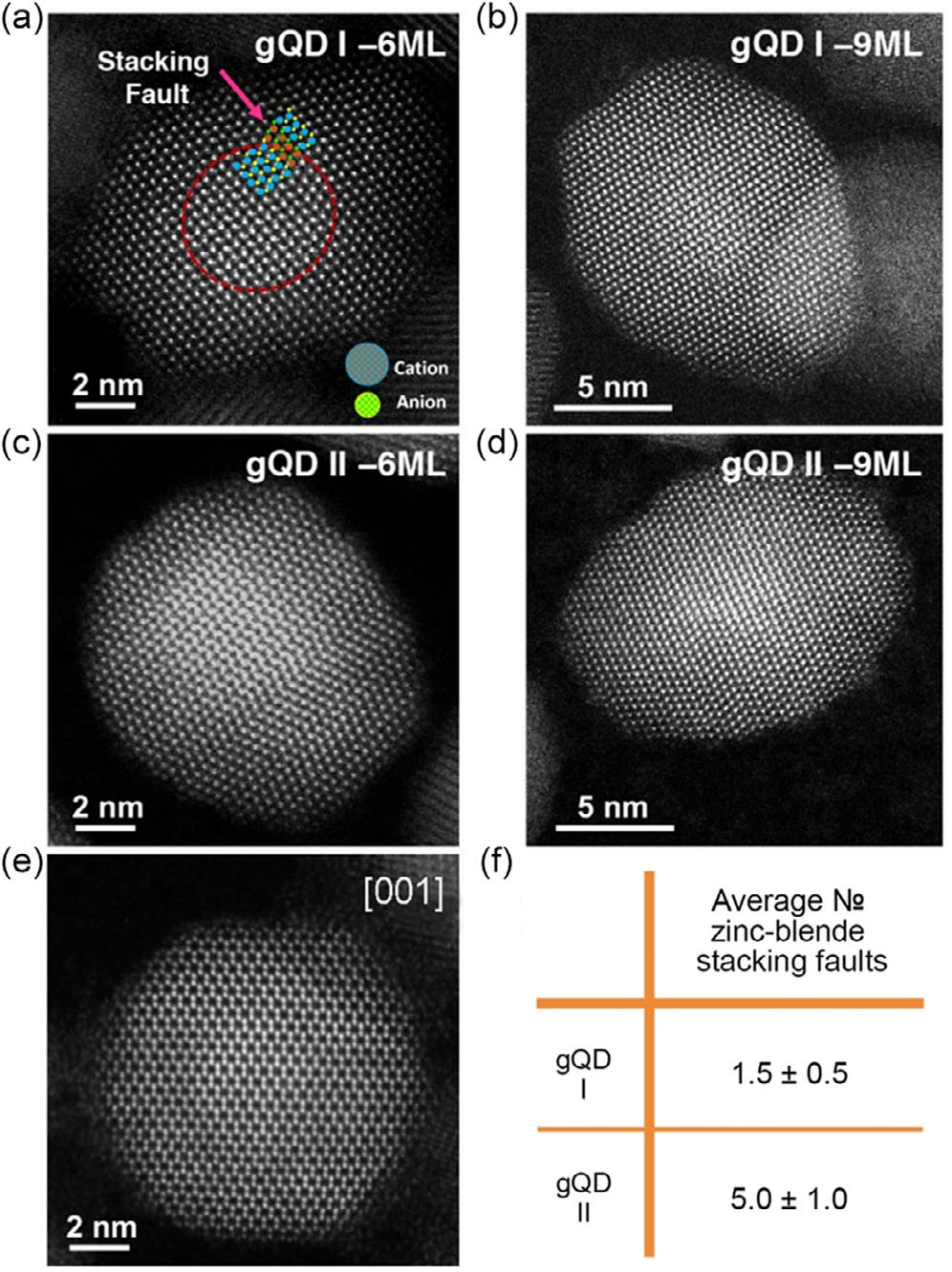
Quantifying defects in the shell. a–d) Aberration‐corrected HAADF‐STEM images of core/shell nanocrystals from the gQD I synthesis possessing ≈6 ML (a) and ≈9 ML (b) CdS shells, and from the gQD II synthesis possessing ≈6 ML (c) and ≈9 ML (d) CdS shells. QDs in (a)–(d) are oriented along the [110] zone axis. e) HAADF‐STEM image of a gQD II nanocrystal oriented along the [001] zone axis, for which stacking faults cannot be visualized. f) Average number of stacking faults observed for each gQD synthesis obtained by analysis 30 and 20 nanocrystals, respectively.

HAADF‐STEM imaging was used to assess the crystallinity of the QDs and to quantify the number of defects present as a function of shell‐growth method. Similar to STEM–EDS, we were not able to use fully “giant”’ nanocrystals. The thick shell (≈5.5–7 nm) would prevent imaging of the core/shell interface with atomic precision, and it would not be possible to distinguish the anions, Se (*Z* = 34) and S (*Z* = 16), present along the same atomic column. 3D electron tomography^[^ ^50^, ^51^, ^52^, ^53^ ^]^ and STEM depth sectioning^[^ ^54^ ^]^ have been used previously in analysis of QDs. However, these techniques require heavy data processing and advanced reconstruction algorithms, allowing only a few QDs to be analyzed. To access a reasonably large sample size and avoid the need for extensive image processing, we employed HAADF‐STEM conducted using an aberration‐corrected instrument for atomic‐scale resolution (see Supporting Information for experimental details). It was found that the thickest shell for which the core/shell interface could be imaged coherently was in the range of 6–9 MLs. An unfiltered high‐resolution HAADF‐STEM image of a 6 ML gQD I nanocrystal is shown in **Figure** **3**a. Both cationic (high contrast) and anionic (low contrast) atomic columns are clearly visible, allowing the probable location of the core (highlighted by a red circle) to be delineated. The highly crystalline core and shell exhibit hexagonal wurtzite crystal structure, and the relatively small lattice mismatch between CdSe and CdS (3.9%) allows for a commensurate interface. Figure S3, Supporting Information, shows the filtered version of this image using a low‐pass band filter to enhance the distinction between core and shell, and to better expose the cation and anion columns. In a similar fashion, 9 ML gQD I and 6 and 9 ML gQD II‐type nanocrystals were imaged and analyzed (Figure 3b,c,d shows representative images). All imaged QDs were monocrystalline without signs of strongly aberrant growth that would lead, for example, to island formation. Nevertheless, in both systems, stacking faults with zinc‐blende (ZB) stacking sequence were present. Stacking faults may participate in relieving interfacial stress resulting even from the relatively small lattice mismatch. We note that to observe the stacking faults the nanocrystals must be oriented along the [110] zone axis. In contrast, imaging along the [001] direction, as shown in Figure 3e, reveals no difference in stacking sequence. These defects may have been missed in previous reports that did not utilize [110]‐oriented nanocrystals.^[^ ^5^ ^]^

Despite a common CdSe core and CdSe/CdS interface, gQD I and gQD II nanocrystals exhibit significant differences in the number of stacking faults that form. Using 30 6 ML gQD I nanocrystals and 20 6 ML gQD II nanocrystals, the average number of stacking faults per QD (Figure 3f) was assessed. gQDs prepared by the SILAR method possessed on average 5 ± 1 stacking faults per nanocrystal, while nanocrystals grown using the HT‐CI method had significantly fewer–only 1.5 ± 0.5, with many QDs being fully defect free (representative defect‐free 9 ML gQD I nanocrystal is shown in Figure 3b). Additionally, we confirmed that the stacking faults begin along the core/shell interface (Figure 3a,c; highlighted with different colors in Figure 3a). As the intensity in HAADF‐STEM images also scales with sample thickness, the presence of a brighter region for both cation and anion columns could be related to an increase in the projected thickness of the sample. However, the intensity in HAADF‐STEM also scales approximately with the atomic number Z^1.7^. Intensity changes, particularly in anion column, may, therefore, also be related to the presence of the CdSe core, i.e., selenium columns are clearly visible. In this way, we used STEM/EDS mapping to confirm the approximate core size and position in the QDs and correlated that with the starting core size. More importantly, as the shell thickness of the gQDs increases to 9 MLs, it is difficult to see the core/shell interface, while the ZB stacking fault is still evident, suggesting that the thinner 6 ML samples are preferred for analyzing the origin of a stacking fault within the nanocrystal, but the 9 ML sample is relevant for confirming that the stacking faults do not “anneal out” during further shell addition, and, instead, remain and extend to the outer limit of the nanocrystal.

To determine whether the stacking faults contribute significantly to the overall phase composition of the nanocrystals, as well as to confirm that the observations made for single nanocrystals by electron microscopy are reflected in a bulk measurement, powder X‐ray diffraction (pXRD) was performed. Analysis of pXRD patterns for both gQD types (Figure S4, Supporting Information) shows that the wurtzite phase dominates the crystal structure. However, Rietveld refinement (see Supporting Information for details) reveals that gQD I and II patterns contain contributions from the ZB phase. Significantly, in agreement with single‐QD analyses, the ZB phase exists to a greater extent in the gQD II nanocrystals than it does in the gQD I nanocrystals—35% compared to 5%, respectively, with the deviation from a pure wurtzitic phase in gQD I being close to nondetectable.

### 2.4 Understanding the Surface

**Figure 4:**
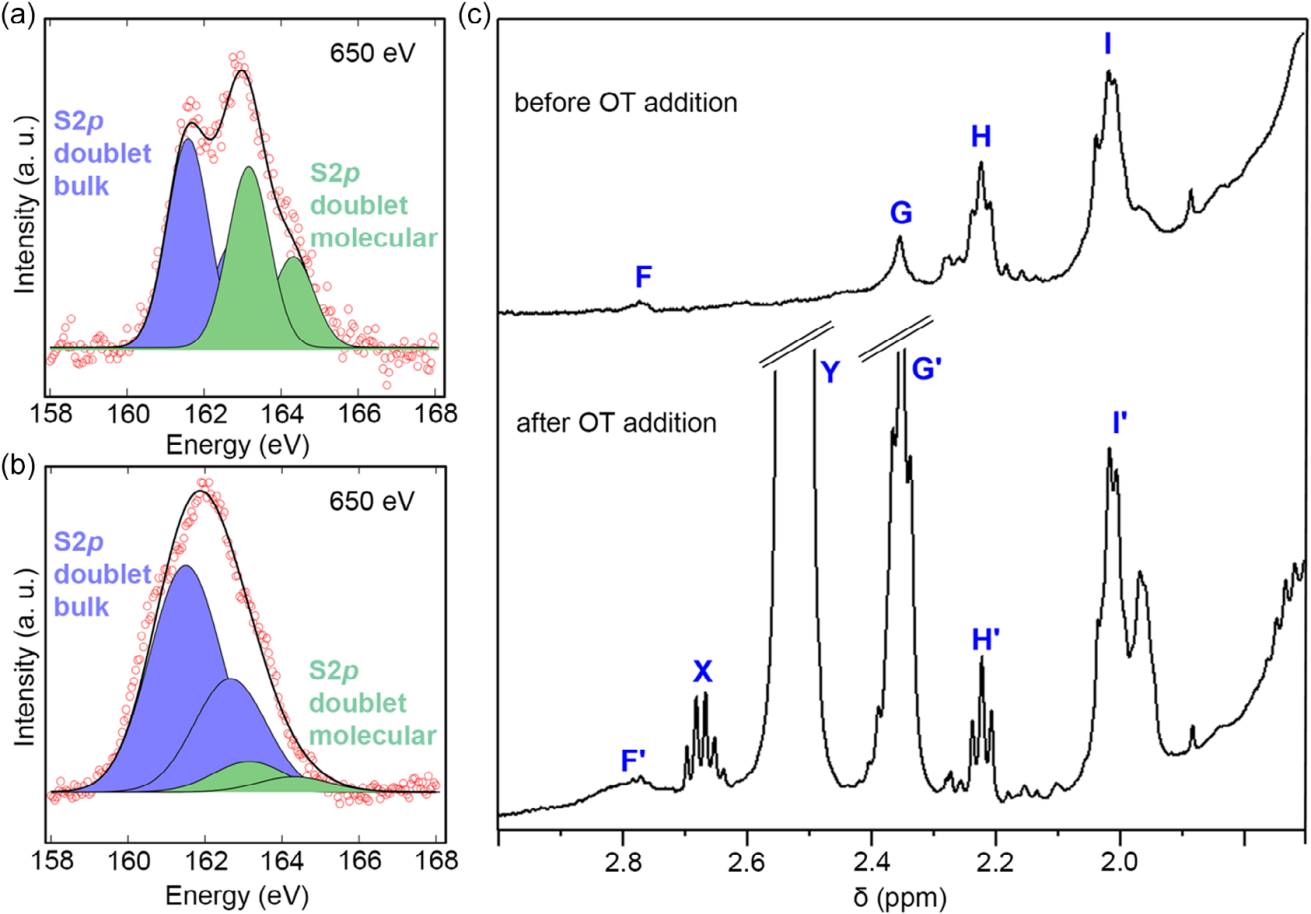
Chemical nature of the gQD surface confirmed. a,b) High‐resolution S 2p spectra for fully “giant” nanocrystals: a) gQD I and b) gQD II. c) ^1^H NMR spectra of gQD I taken in CDCl_3_ before (top) and after (bottom) the addition of octanethiol (OT).

Surface chemical composition of the two gQD systems was assessed by XPS for the full “giant” structures, i.e., ≈15–16 ML CdS shells. In this case, Se does not contribute to the spectra, as it is buried within the thick shell. In the case of gQD I, high‐resolution S 2p spectra fit well with two different spin–orbit split S‐doublets (**Figure** **4**a). The spin–orbit doublet with binding energy of ≈161.5 eV corresponds to S atoms of the bulk crystal structure. The other doublet, having the higher binding energy of >163 eV, is related to S atoms of molecular origin. We attribute these to a thiolate surface ligand (see discussion below and experimental details in the Supporting Information regarding the chemical source of such ligands). The S is likely bonded to a Cd atom but is not simply a surface S atom of the QD crystalline structure. Instead, the observed binding energy matches well^[^ ^55^ ^]^ to a S that is also covalently bound to an adjacent carbon atom (Cd–S–CH_2_–C_*n*_). The greater electronegativity of the C compared to a Cd atom results in a reduction of the negative charge on the S atom, causing a shift to higher binding energies (see Supporting Information for a detailed discussion). In contrast, in the case of gQD II, S binding energies correspond predominantly to the S 2p peak from bulk CdS (S 2p_3/2_ ≈ 161.4 eV) (Figure 4b), with only minor contributions from higher binding‐energy constituents that can be associated with sulfur of some molecular origin (S 2p_3/2_ ≈ 163.1 eV). This result provides strong evidence for the different shell‐growth procedures leading to different surface chemistries.

It has been observed for other nanocrystal systems^[^ ^56^, ^57^, ^58^, ^59^ ^]^ that organosulfur compounds, whether intentionally^[^ ^57^, ^58^, ^59^ ^]^ or unintentionally^[^ ^56^ ^]^ introduced as reactants, can perform dual roles–sulfur source and ligand. The observation of a surface‐bound organothiol ligand on gQD I by XPS suggests that 1‐octanethiol (or thiolate)–which acts as the source of sulfur in the HT‐CI CdS shell growth (gQD I) but not in the SILAR synthesis (gQD II)–is present in the collection of ligands that passivate the QD surface. To substantiate this hypothesis, NMR spectroscopy was employed to interrogate the nature of the ligand composition. gQD spectra were compared with spectra obtained for the likely surface ligands, which included oleic acid and oleylamine for both gQD I and gQD II, as well the suspected surface ligand for gQD I, 1‐octanethiol (see Supporting Information for experimental details). For both gQDs, chemical shifts for oleic acid, oleylamine, and/or their acid–base condensation products (ion pair or amide) are present (Supporting Information Figure S5 and S6, Supporting Information).^[^ ^56^, ^60^, ^61^, ^62^, ^63^, ^64^ ^]^

In the ^1^H NMR spectrum of gQD I, a weak signal (*F*) is present at *δ* = 2.77 ppm (Figure 4c, top) in the region where one would expect the α‐SH_2_ signals to appear,^[^ ^56^ ^]^ shifted downfield relative to the free ligand signal of *δ* = 2.51 ppm (Figure S5, Supporting Information, OT NMR). However, the observation of this signal was inconsistent across a number of samples prepared, possibly indicating that this signal arises from an impurity or that the observation of the weak signal for surface‐bound thiol(ate) is concentration dependent. Prior to ^1^H NMR analysis, samples of gQD I and gQD II were purified by several precipitation–redispersion cycles that significantly reduce the concentration of free ligand in solution and, consequently, bound ligand at the nanoparticle surface. Prior observations of surface‐bound thiol(ate) ligands have indicated weak and broad signals in ^1^H NMR spectra, owing to rapid exchange of surface‐bound thiol(ate) ligands with free thiol in solution.^[^ ^56^ ^]^ The signal *G* shown in Figure 4c (top) is indicative of free OAc, likely in exchange with bound oleic acid on the surface, and *H* is consistent with the α‐CH_2_ signals of another unbound derivative of Olam/OAc (possibly a condensation product or deprotonated salt of OAc). Signal *I*, which presents as a broad, overlapping multiplet, is composed of signals from the allylic protons in ODE, OAc, Olam, and their derivatives.

To confirm whether octanethiol can bind to gQD I if present in adequate concentration in solution, a small amount of octanethiol was added to the suspended gQDs. A broad peak *F′* at 2.769 ppm is observed to appear, along with sharp peaks at *δ* = 2.70–2.65 ppm and *δ* = 2.54–2.50 ppm (*X* and *Y* in Figure 4c, bottom, respectively). The new peak *X* is attributed to the formation of di‐*n*‐octyl disulfide species and not believed to be associated with gQD surface,^[^ ^5^, ^57^, ^65^ ^]^ while *Y* is assigned to free octanethiol. The signal at 2.769 ppm is attributed to the –S–CH_2_– protons of surface bound 1‐octanethiol or 1‐octanethiolate in rapid exchange with free octanethiol. The downfield chemical shift of the bound ligand relative to free ligand is consistent with previous reports of organothiol binding at the surface of PbS QDs. In general, peak broadening compared to the reference peak resonances indicates^[^ ^13^ ^]^ that the species are bound to (or in rapid dynamic exchange with) the nanocrystal surface.^[^ ^56^, ^66^, ^67^, ^68^, ^69^ ^]^ Concomitant with the growth of *F′*, there is an increase in the intensity of the signal corresponding to free OAc (*G′*) likely due to displacement of surface bound OAc/oleate by the added OT.

Taken together with the results from XPS experiments, we can conclude that octanethiol introduced during CdS shell growth by the HT‐CI method can both provide sulfur to the growing nanocrystal and serve as a surface‐bound ligand, likely playing a role in directing crystal growth during synthesis (see discussion below). In this latter function, it can potentially influence the features that are distinct between the two gQD types—crystal shape and aspect ratio, Se–S mixing at the core/shell interface, and/or the prevalence of stacking faults that originate at the interface.

### 2.5 Establishing Synthesis–Structure Correlations

We have shown here that the choice of shell‐growth technique between high‐temperature continuous injection and SILAR methods has a significant impact on gQD structure. This includes the external observables like aspect ratio, as well as the features elucidated here that are buried within the nanocrystal, i.e., interfacial alloying and density of stacking faults. Both gQD I and gQD II nanocrystals are hexagonal bipyramidal in shape, with many nanocrystals exhibiting truncation on one end, indicating exposure of either the 0001 or 000$\bar{1}$ polar basal plane. The employed X‐type (oleate) and L‐type (oleic acid, oleylamine) ligands selectively bind to cationic and neutral facets,^[^ ^70^ ^]^ where octanethiol is an L‐type ligand if protonated and X‐type if deprotonated. Z‐type (neutral electron‐accepting) ligands that could bind to anionic surfaces were not used in either shell‐growth reaction. For these reasons, it is almost certain that the exposed polar plane is the Cd‐terminated {0001} facet, and not an anionic S^2−^‐rich surface. Previously, a detailed structural analysis of similarly shaped CdSe/CdS gQDs showed that the nanocrystal sidewalls are dominated by polar {101$\bar{1}$} facets. In that case, the nanocrystals were thought to be passivated primarily by oleic acid covalently bound via a bidentate interaction to these Cd‐rich surfaces.^[^ ^71^ ^]^ Our XPS and NMR analyses suggest that the surface chemistry is likely more complicated, with contributions also from both the amine and thiol compounds present in the reaction, but given that neither adds a new binding mode (compared to oleic acid/oleate), their presence likely does not strongly influence facet expression. That said, it has been proposed that when thiol is used as the S source, as is the case for gQD I shell growth, the alkyl‐terminated S can become an integral part of the outer surface of the crystal.^[^ ^57^ ^]^ While the potential influence on facet structure is unclear, this mode of thiol incorporation may play an important role in determining QY (see below discussion). Finally, gQD II nanocrystals are more isotropic (Figure 1c). This may be due to the observed significant contribution from the ZB phase, which is of higher crystal symmetry compared to the wurtzite phase.^[^ ^70^ ^]^ It is worth noting that some nanocrystals incorporate sidewalls orthogonal to the {0001} plane, which can result in “bullet”‐shaped nanocrystals. These arise as a result of partial termination of the crystal by the low Miller index, neutral {1$\bar{1}$00} and {11$\bar{2}$0} facets^[^ ^70^ ^]^ or {101$\bar{1}$} facets.^[^ ^72^ ^]^

Even though thiol/thiolate in the role of ligand (L‐ or X‐type, respectively) would not be responsible for eliciting a new crystal shape, its presence along with the higher growth temperature of 310 °C (compared to 240 °C used for the SILAR method) can be a reason for the production of nearly defect‐free shells. First, octanethiol has a relatively low reactivity and as such could better support epitaxial growth and formation of the phase‐pure thermodynamic wurtzite phase.^[^ ^71^ ^]^ Second, as a S source, octanethiol can yield CdS through C–S bond breaking and β‐hydrogen elimination (producing H_2(g)_ and octene_(g)_), but thermal decomposition takes place at 400 °C, which is above the shell‐growth reaction temperature. Likely, this process at lower temperature is mediated by the QD surface, such that octanethiol exists in solution throughout the growth process, giving it an opportunity to also function as a ligand. In this case, we assume that prior to decomposition surface‐bound R′S– and solvated R′SH are in equilibrium. This provides a mechanism for reversible addition of –S and, thereby, an enhanced opportunity for surface‐atom rearrangement that can serve to heal defects before they are frozen into the crystal. This pathway is absent in SILAR growth.

Unlike the case for crystalline phase and aspect ratio, ligands do not appear to play a principal role in determining the alloy nature of the core/shell interface. A control experiment exposed, instead, the important function of reaction time in inducing interfacial alloying. For the gQD II SILAR process, we utilize long anneal steps in between each addition of precursor. Specifically, after addition of cationic precursor for a given monolayer, the reaction is held at the reaction temperature for 2.5 h, while after addition of anionic precursor the reaction is held for 1 h. Using such “long cycle” SILAR means that QDs are exposed to 240 °C for >50 h. To assess the effect of long anneal times on interfacial alloying, we resuspended gQD I nanocrystals in a high‐boiling solvent and subjected them to a temperature of 240 °C for an amount of time (≈38 h) that when combined with their own growth time of ≈14.5 h was equivalent to the duration of a long‐cycle SILAR reaction. In this case, the radial extension of Se increased from 0.50 ± 0.20 nm (Figure 2) to 1.27 ± 0.22 nm (Supporting Information Figure S7a,b and Table S1, Supporting Information). Thus, a long anneal time alone can induce interfacial alloying. And, once again, EDS results are supported by ensemble XPS analysis. Using the 1080 eV incident photon energy in an analysis of annealed gQD‐I 9 ML nanocrystals, we find a change in observed Se from 12.4% to 31.3% (Figure S7c, Supporting Information). Significantly, comparative analysis by HRSTEM of gQD I nanocrystals pre‐ and postanneal yielded no difference in the quantity of interface defects (ZB stacking faults; Figure S7d, Supporting Information), which suggests that these defects are “baked in” during shell growth and are a direct result of the choice of ligand/precursor chemistry and cannot be easily annealed out.

In summary, several correlations between synthesis parameters and nanocrystal structure can now be asserted (**Table** **1**). First, the degree of interfacial alloying can be enhanced by increasing the time QDs spend at elevated temperature (here, ≥240 °C). The high‐temperature (310 °C) continuous injection method does not, itself, provide the necessary conditions for extensive alloying. In addition, reaction chemistry is a strong driver in determining the density of ZB stacking faults, with the larger number of stacking faults observed by HAADF‐STEM imaging in SILAR‐grown QDs translating to significant ZB content in pXRD patterns. Finally, octanethiol, when employed at the high growth temperatures used for the HT‐CI method, functions as both the sulfur source and a postgrowth surface ligand. During synthesis, this dual role likely contributes to the observed enhanced crystalline quality of the resulting gQDs.

**Table 1:** Summary of synthesis–structure correlations for CdSe/CdS gQDs prepared by continuous injection (HT‐CI; gQD I—before and after postsynthesis anneal) or SILAR (gQD II) shell‐growth methods

Structural feature	HT‐CI: gQD I	Long‐cycle SILAR: gQD II	gQD I + postsynthesis anneal
Prominent interfacial alloying	−	+	+
Low defect density in shell	+	−	+
Organosulfur as surface ligand	+	−	Not assessed

### 2.6 Connecting Structure to Function

gQD I and gQD II nanocrystals also differ in how they function as light‐emitting nanomaterials, suggesting that the observed synthesis‐induced variations in structural properties can be further correlated with differences in optical properties. The performance characteristics we survey here are: photoluminescence QY, single‐gQD photostability under “stress”, i.e., at elevated temperature under constant photon flux for long exposure times (>1 h), and relative efficiency of nonradiative Auger recombination. Room‐temperature blinking and photobleaching behavior could also be considered, but, as shown above (Figure 1d,e), these parameters show little variation between the two QD materials, with both performing as desired for a gQD—essentially nonblinking and nonphotobleaching over 1 h observation.

#### 2.6.1 Quantum Yield

**Figure 5:**
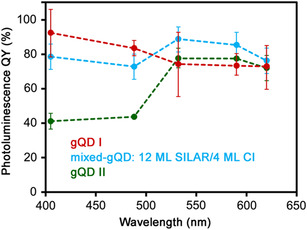
gQD QY as a function of excitation wavelength and shell‐growth method.

gQD I outperforms gQD II in emission efficiency, establishing a correlation between synthesis by HT‐CI and high QY. However, of the three identified differences in nanocrystal structure—interfacial alloying, defect density, and surface ligand chemistry—which most strongly influences QY? Here, to establish the correct structure–function correlation, we compare the wavelength‐dependent QYs for gQD I, gQD II, and a gQD prepared by growing the first 12 MLs of CdS using SILAR and the final 4 MLs using continuous injection (**Figure** **5**). Using excitation wavelengths (*λ*_exc_) from 405 to 625 nm, we selectively produce excitons either in the CdSe core (*λ*_exc_ > 515 nm) or in both the CdSe core and the CdS shell (*λ*_exc_ < 515 nm). Notably, in the latter case excitons form primarily in the shell as CdS represents >95% of the particle volume. We find that when the QDs are excited at 405 nm, gQD I exhibits near‐unity QY (>90%), while the QY for gQD II is approximately half that (<45%). A similar result is obtained when the gQDs are excited just below 500 nm. However, when an *λ*_exc_ of 532 nm is used, the QY for gQD II almost doubles to ≈80%. This remarkable *λ*_exc_‐dependent increase in PL QY is maintained as the *λ*_exc_ is shifted further to the red, toward the bandgap of the CdSe core (CdSe 1S = 610 nm). The fact that bluer excitation energies, which are capable of generating excitons in the CdS shell, lead to lower QYs in the case of gQD II implies that these gQDs are susceptible to nonradiative carrier relaxation mediated by either the shell or the surface, while gQD I is less impacted by such processes. And, when only the CdSe core is excited, gQD I and gQD II have similar, near‐unity QYs.

The QY results for gQD I and gQD II alone are not complete, as they do not distinguish between the different processes that could lead to lower QY in the case of gQD II. Structural defects in the shell, like the observed ZB stacking faults, have been cited as a possible cause of reduced QYs in related CdSe/CdS heterostructures, such as dot‐in‐nanorods.^[^ ^73^ ^]^ However, this conclusion is controversial as others have observed that lattice defects do not necessarily lead to diminished QY, pointing instead to electronic traps at the CdS–ligand interface.^[^ ^74^ ^]^ To help distinguish between these two possibilities, we prepared a third type of gQD—one synthesized using the SILAR method for growth of the first 12 CdS monolayers followed by HT‐CI for addition of the final four shell monolayers. These “mixed‐synthesis” gQDs have a higher density of stacking faults in the shell as evidenced by a significant ZB contribution to the pXRD pattern, similar to gQD II nanocrystals (Figure S8c, Supporting Information), but a surface chemistry more like gQD I nanocrystals, i.e., likely terminated in part by thiol ligands. Compellingly, when excited at 405 nm these QDs exhibit high QYs like their gQD I counterparts (Figure 5). They also show enhanced QYs upon transition to core‐only excitation, similar to gQD II behavior, but as their starting QY is already close to unity (≈80%), the magnitude of the increase is smaller (≈10% increase compared to gQD II's ≈100% increase). Thus, with behavior most like gQD I, with which it shares ligand composition, results for the mixed‐synthesis gQD suggest that surface chemistry plays the larger role in determining QY.

According to high‐resolution XPS results, the gQD I surface is characterized by the significant presence of S bound to C (or other more electronegative element). In contrast, gQD II lacks substantial coordinated organothiol, which may act to stabilize undercoordinated surface S in gQD I. Unsaturated S is a known source of hole traps, which has been reported previously for metal chalcogenide nanocrystals passivated solely by carboxylic or amine X‐ and L‐type ligands.^[^ ^75^, ^76^, ^77^ ^]^ Hole trapping is an ultrafast process (<10 ps) and can compete with ultrafast carrier relaxation to core band‐edge states.^[^ ^78^ ^]^ Although we cannot conclusively identify the mechanism by which octanethiol can serve to eliminate undercoordinated surface S states, its apparent ability to do so is likely a primary driver for high QYs in gQD I‐type nanocrystals. Additionally, we conclude that the presence of stacking faults or associated interfacial strain is not inherently limiting for achieving high QY, although this structural feature may not be completely innocent, as evidenced by the small enhancement (≈10%) in QY for the mixed‐gQD system when shell excitation is avoided (excitation wavelengths >515 nm; Figure 5 light blue data).

#### 2.6.2 Long‐Term Single‐Nanocrystal Photoluminescence Stability under High Photon Flux and Elevated Temperature

In addition to QY, stability of emission is a critical performance criterion for many QD applications. For example, reliability over time is required for delivering single photons on‐demand in envisioned single‐photon sources, as well as for delivering consistent lumens per watt and light color over time for QD‐enabled white‐light LED bulbs. In these scenarios, the emitters can experience ambient levels of oxygen and humidity, elevated temperature, and constant and/or intense photon fluxes. Under these conditions, even gQDs will fail given enough time. We have shown previously that gQD I and gQD II eventually succumb via two distinct photobleaching processes—abrupt failure by hot‐carrier trapping and/or gradual dimming by accumulation of charge.^[^ ^20^, ^79^ ^]^ The former mechanism is more characteristic of gQD I emitters and results in ensemble photobleaching that is accompanied by a decrease in the total number of emissive gQDs, while the latter is more prominent in gQD II nanocrystals and results in ensemble bleaching for which the total number of emissive gQDs remains constant, but each nanocrystal emits at a reduced intensity (dimming) from states of increasing excess charge.^[^ ^20^ ^]^

Although these two primary mechanisms for gQD bleaching are now understood, the structural characteristics responsible for the distinct behaviors of the differently synthesized gQDs have yet to be elucidated. Based on the results here, we can conclude that gQD I and gQD II differ in multiple structural features, which complicates the effort to correlate function with structure. Like the case for QY, we turn to the “mixed‐synthesis” strategy in an attempt to isolate the primary structural feature responsible for a specific photobleaching pathway. To this end, we prepared gQDs for which the first 4 or 8 MLs were grown by HT‐CI, and the remaining shell layers were grown by the SILAR method. In this case, the core/shell interface is most like CI‐prepared gQDs, with little alloying and low defect density in the initial stages of shell growth, while the surface chemistry would mimic SILAR‐prepared gQDs.

**Figure 6:**
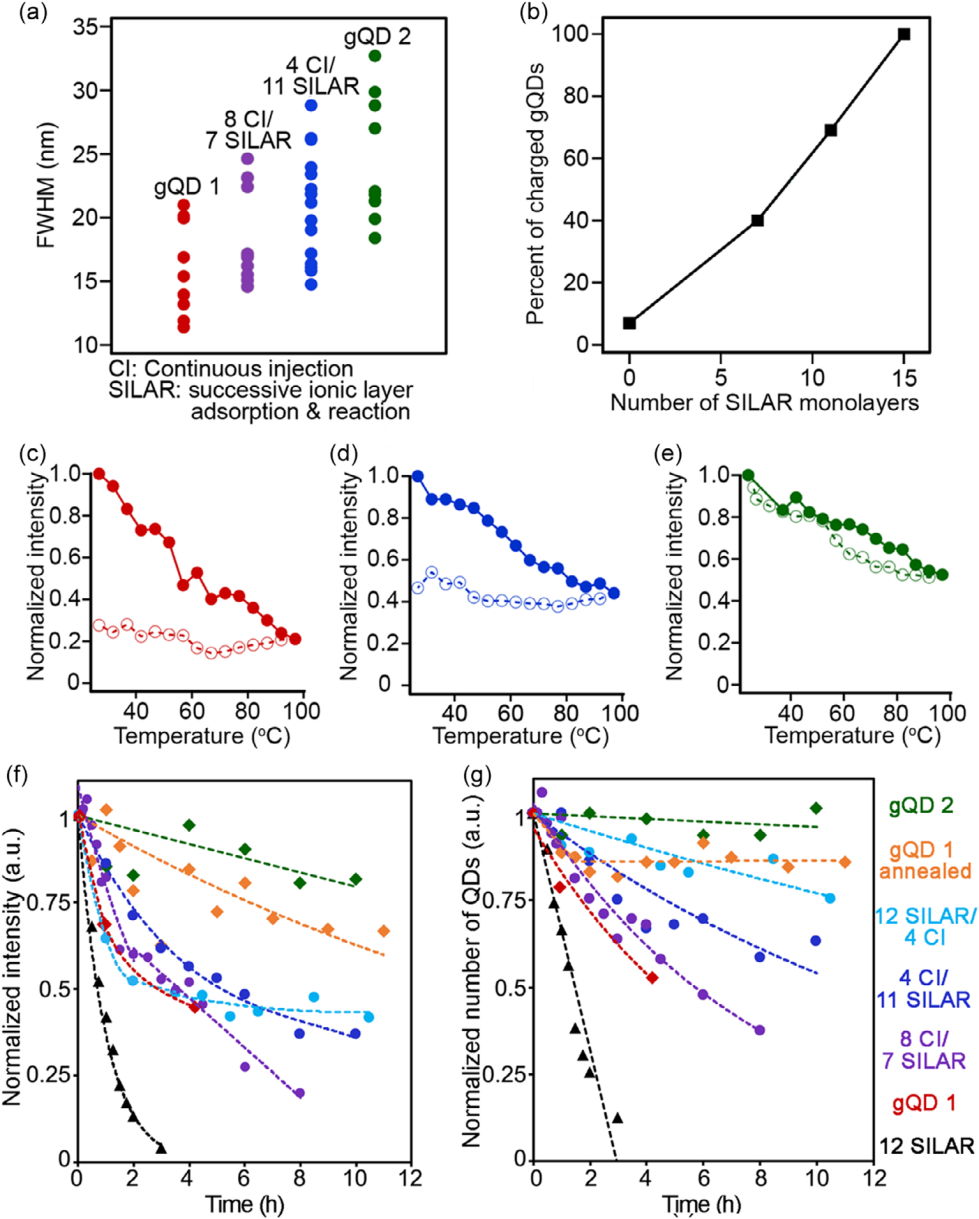
Characteristics of gQDs across a mixed CI‐SILAR synthesis series. a) Room‐temperature homogeneous line widths (FWHM values) obtained for single‐gQDs representing different shell‐growth methods. b) Dependence of observation of photocharging in gQDs as a function of the number of shell MLs (number out of 15; obtained by analysis of FLID plots as shown in Figure S9, Supporting Information) that are prepared using the SILAR method (with balance obtained by CI method). c–e) Response of different gQDs to temperature and ultrahigh photon flux (15 W mm^−2^): photoluminescence intensity as a function of temperature (solid circles correspond to increasing temperature, open circles to decreasing temperature) for the gQD shell‐synthesis series (colors match (b)). f,g) Long‐term photobleaching behavior under thermal (≈100 °C) and photon (1 W mm^−2^) stress for products of the shell‐growth series: f) average single‐gQD intensity (normalized) over up to 11 h and g) fraction of gQDs that remain emissive over time. Dashed lines are guides to the eye.

Interestingly, the gQDs prepared by mixing‐and‐matching the shell‐growth techniques possess a number of “mixed” characteristics. First, ZB stacking faults clearly appear during the SILAR growth stage, as pXRD patterns again show unmistakable contributions from the ZB phase (Figure S8a,b, Supporting Information). Furthermore, photoluminescence full‐width‐half‐maxima (FWHM) obtained from single QDs from each synthesis reveal a trend toward increasing peak width with increasing numbers of shell monolayers grown by SILAR (**Figure** **6**a). Finally, we have previously reported that SILAR‐gQDs emit mostly from charged (trion or higher order) excited states, while HT‐CI‐gQDs emit almost exclusively from pure exciton states.^[^ ^27^ ^]^ Here, we apply the technique—fluorescence lifetime intensity distribution (FLID) mapping—to assess the extent of charged‐state emission in the case of the mixed‐synthesis gQDs. We find a clear trend of increasing charged‐state emission with increasing number of SILAR monolayers (Figure 6b; see Supporting Information for details of the FLID experiments and Figure S9, Supporting Information).

The question, then, remains—how do these trends and the now understood structural features map to photo and thermal stability? Initially, we compare gQD I, gQD II, and a mixed‐synthesis gQD in a simple heat‐up/cool‐down experiment (Figure 6c,d,e). All three gQDs become less emissive at elevated temperature, but percent‐loss is less with increasing SILAR‐shell content. Similarly, recovery trajectories depend on SILAR‐shell content, with gQD II recovering 100% of its original intensity, and both gQD I and the mixed‐synthesis gQD remaining at their respective depressed high‐temperature intensities.

In a more elaborate photobleaching experiment, we compare the full suite of mixed‐synthesis gQDs, as well as gQD I and gQD II, in long‐term photobleaching experiments during which emission from individual nanocrystals is tracked over time at elevated temperature under continuous laser excitation (Figure 6f,g). The mixed‐synthesis gQDs prepared using an ≈50/50 split between the two techniques (8 CI/7 SILAR) exhibit gQD I‐like photobleaching behavior—significant decline in PL intensity with time (Figure 6f; compare red and purple trajectories) that is associated with a clear reduction in the fraction of gQDs that remain emissive (Figure 6g). The mixed gQDs dominated by SILAR‐grown shells (4 CI/11 SILAR and 12 SILAR/4 CI) also show marked intensity decline (Figure 6f; compare dark blue and light blue trajectories); however, compared to gQD I a higher fraction of gQDs remain emissive over time (dimming but not quenching). This is especially true in the case of 12 SILAR/4 CI gQDs, which, interestingly, shares positive traits with both types of gQD—the higher QY of gQD I (Figure 5) paired with the high retention of emissive QDs of gQD II (Figure 6g). As discussed above, the tendency to dim over time rather than to stop emitting altogether is a gQD II trait.^[^ ^20^ ^]^ Thus, with respect to photobleaching mechanism, these mixed gQDs dominated by SILAR‐grown shells more closely resemble the all‐SILAR nanocrystals. Based on these results, we can conclude that an as‐yet unidentified SILAR‐induced trait positively impacts the ability of a gQD to remain at least partially emissive over time under photo/thermal stress. Unlike the case for QY, the key trait is unlikely to be surface chemistry because these high‐SILAR mixed gQDs terminate with different surface ligands, ending in either SILAR or HT‐CI fashion.

To ascertain which remaining feature—alloying and/or defect density—is responsible for the observed behavior, we add an additional gQD to the analysis. gQD I nanocrystals that were annealed postsynthesis were also subjected to the long‐term stability test. As discussed above, this treatment has the effect of increasing interfacial alloying but not the density of defects in the shell. Interestingly, we find that gQD I's stability increases significantly following annealing (Figure 6f; compare orange trajectory with original red trajectory), and the fraction of emissive QDs over time (≈1–12 h) is approximately constant after an initial decrease (Figure 6g), very much like gQD II nanocrystals (green trajectory). The primary change to the nanocrystals upon annealing is the degree of alloying (Figure S7, Supporting Information). Thus, we surmise that this gQD feature is the key structural characteristic responsible for long‐term stability and the ability to remain emissive, albeit from dimmer, charged excited states. That said, we note that this result should be viewed in the context of the shell needing to be sufficiently thick. Namely, if the CdS shell is too thin, long‐term stability under photo and thermal stress is not guaranteed even if the shell‐growth method is long‐anneal SILAR. This is event in the comparison of a CdSe/CdS gQD comprising only 12 monolayers of CdS shell (Figure 6f,g black trajectory) with all other QD subjects.

#### 2.6.3 Auger Recombination

**Figure 7:**
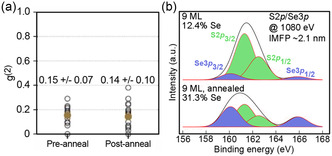
Effect of annealing on *g* ^(2)^ values for gQD I‐type nanocrystals. a) *g* ^(2)^ values pre‐ and postanneal. The CdS shell is ≈9 ML thick and grown using the continuous‐injection method. b) XPS spectra for 9 ML CdSe/CdS core/shell QDs prepared by HT‐CI pre (top) and post (bottom) anneal, showing the doublet peaks for S 2p and Se 3p orbitals obtained using a photon energy of 1080 eV.

Finally, we consider a third key attribute of gQD optical performance—suppression of nonradiative Auger recombination and enhancement of biexciton efficiency. We showed previously that there is no significant difference in Auger suppression between gQD I and gQD II, as indicated by their having very similar biexciton QYs (≈30%).^[^ ^20^ ^]^ This was determined by comparing the second‐order photon‐correlation function, *g* ^(2)^, for a representative sample of nanocrystals from each system. *g* ^(2)^ values provide a measure of biexciton QY, and similar values indicate a similar degree of Auger suppression. **Figure** **7**a shows the measured *g* ^(2)^ values for a series of gQDs from a 9‐ML gQD I‐type synthesis and for the same nanocrystal system following annealing (see Supporting Information for experimental details). The *g* ^(2)^ values are similar pre‐ and postanneal, indicating that there are no significant differences in the level of suppression of nonradiative Auger recombination of the biexciton. Importantly, based on the assessment presented here demonstrating first that gQD I and gQD II nanocrystals are characterized by marked differences in the extent of interfacial alloying and second that annealing of gQD I nanocrystals increases Se–S alloying (corroborated in Figure 7b analysis of XPS spectra), we surmise that the absence of differences in *g* ^(2)^ values for either compared system (gQD I vs gQD II^[^ ^20^ ^]^ and preanneal gQD I and postanneal gQD I) indicates that interfacial alloying of the anions is not a key factor leading to Auger suppression. This is in contrast with previous literature that has reported a causal link between alloying and mediation of Auger processes.^[^ ^80^ ^]^ Instead, our detailed analysis of the actual Se–S interfacial mixing (STEM–EDS and energy‐dependent XPS) leads us to a different conclusion, namely, that the similar *g* ^(2)^ behavior derives from the common gQD features of a thick shell and a quasi‐type‐II electronic structure.

## 3 Conclusion

Using a series of single‐nanocrystal and ensemble structural and compositional analysis techniques, we have systematically assessed the nature of the prototype gQD—thick‐shell CdSe/CdS core/shell QDs—from the inside out. By “looking under the hood” we have been able to: 1) identify differences in structure and/or composition at the core/shell interface, within the shell, and at the shell surface between two gQDs synthesized by distinct shell‐growth methods (HT‐CI and SILAR), and 2) establish structure–function correlations that result from these differences. The elucidated structure–function correlations are summarized in **Table** **2**. The strongest positive correlations were between interfacial anion alloying and long‐term photostability under device‐relevant photon flux (1–10 W mm^−2^) and elevated temperature (100 °C), as well as enhanced passivation of surface S atoms and high QY. The presence of ZB stacking faults do not themselves support or prevent high stability or high QY. The mechanism of photobleaching is likely influenced by both interfacial alloying and the nature of surface passivation. Namely, all else being equal, an increase in interfacial alloying leads not only to a significant enhancement in emission‐intensity stability over time, but causes the primary photobleaching mechanism to switch from abrupt failure to dimming. Finally, in contrast with some previous reports, more extensive interfacial alloying does not, in fact, lead to enhanced Auger suppression/biexciton QY. Taken together, the results address outstanding questions pertaining to the novel behavior of this class of QD and provide a clearer path toward the design of further optimized high‐QY, nonblinking, and truly robust QD emitters for applications from single‐photon sources to solid‐state lighting.

**Table 2:** Summary of structure–function correlations

Functionality	Significant interfacial alloying	Minimal interfacial alloying	High defect density	Low defect density	Thiol surface ligand
High QY	Na)	N	N	N	+
High stability to photobleaching	+b)	−	N	N	N
Auger suppressed	N	N	N	N	N
Photobleaching mechanism					
Gradual dimming	+	−	N	N	N
Sudden on → off	−c)	+	N	N	N

a) N: neutral association;

b) +: positive association;

c) −: negative association.

## 4 Experimental Section

Experimental details are provided in the Supporting Information.

## Conflict of Interest

The authors declare no conflict of interest.

## Supporting information

Supplementary Material

## Data Availability

The data that support the findings of this study are available from the corresponding author upon reasonable request.
