# Supplementary material for: From Inside Out: How the Buried Interface, Shell Defects, and Surface Chemistry Conspire to Determine Optical Performance in Nonblinking Giant Quantum Dots
Source: Small Sci. 2023 Oct 10;3(11):2300092. doi: 10.1002/smsc.202300092 (PMC11935896; doi:10.1002/smsc.202300092)
Supplement: Supplementary file 1 — Supplementary Material [file SMSC-3-2300092-s001.pdf]

SUPPORTING INFORMATION

From Inside Out: How the Buried Interface, Shell Defects and Surface Chemistry Conspire to Determine Optical Performance in Non-Blinking Giant Quantum Dots

*Ajay Singh,[1] Somak Majumder,[1] Noah J. Thompson Orfield,[1] Ibrahim Sarpkaya,[1,†] Dennis Nordlund,[2] Karen C. Bustillo,[3] Jim Ciston,[3] Victoria Nisoli,[1] Sergei Ivanov,[1] Eric G. Bowes,[1] Han Htoon,[1] Jennifer A. Hollingsworth[1]**

[1]Materials Physics & Applications Division: Center for Integrated Nanotechnologies, Los Alamos National Laboratory, Los Alamos, New Mexico 87545, United States
[2]Stanford Synchrotron Radiation Light Source, Stanford, California 94309, United States
[3]National Center for Electron Microscopy, Molecular Foundry, Lawrence Berkeley National Laboratory, 1 Cyclotron Road, Berkeley, California 94720, United States
[†]Present address: Bilkent University UNAM – National Nanotechnology Research Center, Ankara 06800, Turkey

Table of Contents

Materials and Synthetic Methods……………………………………………………………..2
STEM-energy dispersive X-ray spectroscopy (STEM-EDS) elemental mapping………..5
Aberration-corrected STEM……………………………………………………………..5
Energy-dependent X-ray photoelectron spectroscopy…………………………………....6
Powder X-ray diffraction (XRD) analysis………………………………………………...9
NMR analysis………………………………………………………………………….....10
Single-QD photoluminescence microscopy and spectroscopy, including $g^{(2)}$………….. 14
Statistical analysis………………………………………………………………………..16
Figure S1………………………………………………………………………….........17
Figure S2………………………………………………………………………….........19
Table S1………………………………………………………………………….........20
Figure S3………………………………………………………………………….........21
Figure S4………………………………………………………………………….........22
Figure S5………………………………………………………………………….........23
Figure S6………………………………………………………………………….........24
Table S2………………………………………………………………………….........25
Table S3………………………………………………………………………….........26
Table S4………………………………………………………………………….........27
Figure S7………………………………………………………………………….........28
Figure S8………………………………………………………………………….........29
Figure S9………………………………………………………………………….........30
Supporting References…………………………………………………………………..31

Experimental

Materials

Cadmium oxide (CdO, 99.99%), oleic acid (OAc, 90%), 1-octanethiol (OT, 98%) and selenium (1–3 mm shots, 99.99 %) were purchased from Alfa Aesar. Sulfur (S_8, 99.99%) and 1-octadecene (ODE, 90%) were purchased from Acros Organics. Trioctylphosphine (TOP, 97%) trioctylphosphine oxide (TOPO, 99%) and oleylamine (OAm, 70%) were purchased from Sigma Aldrich, octadecylphosphonic acid (ODPA, 97%) was purchased from Strem and used without further purification.

Methods

Synthesis of CdSe cores: CdSe quantum dot (QD) cores were synthesized by previously published literature protocol with minor modifications.[1] In a typical synthesis, CdO (60 mg), TOPO (~3 g) and ODPA (280 mg) were combined and degassed (under vacuum) for 1 h at 110 °C. Following the degassing, the reaction mixture was heated to 325 °C under Ar atmosphere, when the dissolution of CdO in phosphonic acid is observed (starting at ~300 °C), associated with a color change of the mixture from dark reddish brown to optically clear, colorless; indicating the formation of Cd-ODPA complex. At this point, 1 ml of TOP was injected and the reaction mixture was heated to 380 °C. When the temperature stabilized, Se (60 mg) dissolved in TOP (0.5 mL) prepared separately in glove box was rapidly injected into the reaction mixture to induce nucleation of CdSe cores. After 1.5 min of growth of the CdSe QD cores, the heating mantle was promptly removed and the reaction was allowed to cool down to room temperature. To prevent solidification of TOPO, ~2 mL anhydrous hexane was added when the temperature reached 60 °C. The crude core solution was transferred to glovebox via septa caped vials purged with Ar.

Purification of QDs from excess ligands and unreacted precursors was performed by multiple (2-3) ethanol precipitation – centrifugation - hexane redissolution cycles inside the glove box. Final QDs were dispersed in hexane and stored for future use under inert atmosphere. Slight variation in the annealing time post Se-TOP injection (1 min 30 second to 2 mins) yielded wurtzite CdSe cores with first absorption maxima ranging from 600 to 610 nm (core diameter ~ 5 nm) used for a series of core shell syntheses described below.

Preparation of stock solutions: 0.2 M solution of elemental S_8 dissolved in ODE(S_8/ODE) and 0.2 M Cd-oleate (by dissolving CdO in oleic acid) were prepared and stored under standard Schlenk line conditions (Ar atmosphere). Two distinct molar ratios of 0.2 M Cd-oleate viz. [Cd]:[oleate] = 1:4 and 1:10 was prepared and used for the syntheses. For the synthesis of gQD I (see below), 0.1 M Cd-oleate was prepared by diluting as prepared 0.2 M Cd-oleate inside the glovebox with requisite amount of ODE; 0.12 M solution of OT in ODE was prepared and stored in the glovebox.

Synthesis of giant quantum dots (gQDs): CdSe/CdS gQDs were synthesized by two shelling protocols. The synthesis of gQD I was carried out by the "continuous injection" process, reported in recent literature with minor modifications.[2] gQD II was synthesized by the SILAR process following the procedure described in Ref. 3

gQD I: In a typical synthesis, 1.0 x 10^{-7} mol of CdSe cores were mixed with 3 mL of OAm and 3 mL of ODE in a 250 mL four necked flask and degassed for 0.5 h at room temperature and 1 h at 80 °C. Following degassing, the reaction mixture was heated to 310 °C at the rate of 15 °C/min under Ar, during the course of which Cd-oleate (0.1 M, 5 mL) and OT (0.12 M in ODE, 5 mL) were simultaneously injected at the rate of 3 mL/h starting at 240 °C. On completion of shell precursor addition, the reaction mixture was annealed at 310

°C after swift injection of 1 mL of OAc for 1 h and then cooled down to 90 °C for degassing for 0.5 h. The degassing step was found to be highly effective in circumventing the problem of violent bumping of the reaction mixture (probably resulting from volatile octane, octene side products from cleavage of OT) that is observed during shell growth above 6 MLs. Following degassing, the reaction mixture is again raised to 310 °C, in the manner described above with shell precursor infusion starting at 240 °C. In this way, multiple injections of 5 mL portions of Cd-oleate and OT were performed to yield the desired CdS shell thickness (15 MLs). 0.1 M Cd-oleate ([Cd]:[oleate] = 1:4) was employed for shell growth until 6 MLs, while 0.1 M Cd-oleate ([Cd]:[oleate] = 1:10) was used for 6-15 MLs. The synthesized gQDs were precipitated from growth solution by ethanol and redissolved in hexane.

gQD II: Briefly, 2.0 x 10^{-7} mol of CdSe cores were combined with 5 mL OAm and 5 mL of ODE and degassed at 120 °C under vacuum for 1 h. Following degassing, the reaction temperature was raised and maintained at 240 °C under Ar atmosphere. Alternate injections of 0.2 M S_8/ODE solution and 0.2 M Cd-oleate were performed in dropwise fashion with a syringe, allowing an annealing time of 1 h following each S addition and 2.5 h following each Cd addition. The volume of precursors delivered was calculated so as to yield 1 ML of CdS (~0.34 nm) after each addition cycle. 15 MLs thickness (>5 nm) of CdS were epitaxially grown on the CdSe cores in this manner. 0.2 M Cd-oleate ([Cd]:[oleate] = 1:10) was employed for shell growth. The gQDs were precipitated from growth solution by ethanol and redissolved in hexane. For synthesis of gQDs in control experiments with lower anneal times, 15 mins annealing was allowed in between alternate 0.2 M S_8/ODE solution and 0.2 M Cd-oleate injections, while all other parameters were kept the same.

Annealing gQD I: Anneal sample was prepared by annealing unwashed growth solution (0.8 mL) of gQD I nanocrystals in 3 mL ODE for 38 h to make annealing time at par with gQD II synthesis conditions (16 ML gQD II sample). Therefore, QD washing was not performed and no additional ligands were added prior to annealing. Thus, residual ligands present in growth solutions, e.g., oleylamine, oleate, thiolate, were present in the medium.

STEM-energy dispersive X-ray spectroscopy (STEM-EDS) elemental mapping: STEM-EDS mapping were acquired using FEI-TitanX equipped with a Bruker SuperX quad EDS detector with 0.7 srad collection angle specified. The accelerating voltage was 200 kV and incident beam current was 740 pA. The EDS map pixel size was 0.3 nm and the cumulative dwell time per pixel was about 10 ms. STEM images before and after EDS mapping were acquired to ensure that no visible sample damage was evident.

Aberration-corrected STEM: FEI TEAM I and TEAM 0.5 instruments were used to provide HAADF-STEM atomic resolution images at 300 kV accelerating voltage. The probe semi-convergence angle was 17 mrad. To ascertain the true nature of the interface coherently, it was necessary to study gQDs when only a few monolayers of shell material were present. STEM resolution is impeded with increasing thickness. I.e., at 16 MLs (diameter of 18 ± 1.5 nm) the QDs possess >5 nm of CdS shell. This extra thickness obfuscates the core-shell interface, making true gQDs unsuitable candidates for the purpose of TEM imaging of the interface. Consequently, the 6 ML and 9 ML core/shell QDs were analyzed.

Energy-dependent X-ray photoelectron spectroscopy: The synchrotron XPS was performed at BL 10-1 of Stanford Synchrotron Radiation Lightsource (SSRL) using a ring current of 500 mA and a 1000 l.mm-1 spherical grating monochromator, operated with 40 um entrance and exit slits. A cylindrical mirror analyzer (CMA) was used with a pass energy of 25 eV (for higher resolution spectra, <0.4 eV total resolution) and 50 eV (for intermediate resolution spectra, <0.7 eV total resolution) to record the photoemission spectra at two different incidence energies, 650 eV and 1080 eV. All spectra were recorded in the horizontal plane with the CMA mounted perpendicular to the incident photon beam with a grazing incidence angle of 20° relative to the sample. The spectra were normalized by the current from a gold-evaporated fine grid that is positioned upstream of the main chamber.

Samples for XPS measurements were prepared as large-area continuous nanocrystal monolayers, and the QDs were transferred to undoped single crystal silicon substrates to avoid chemical doping from substrate and to avoid electron beam charging during the measurements. All samples were mounted on an aluminum sample holder with double-sided carbon tape in an argon-filled glove box, and transferred in a double-contained jar to a glove bag purged with argon connected to the XPS load-lock chamber. All data were acquired under ultrahigh vacuum (10^{-9} Torr) in a single load at room temperature.

All spectra were treated similarly, with the binding energy scale referenced to the Au4$f_{7/2}$ peak (84.0 eV) from a pure gold sample. Some samples experienced small shifts due to charging, so all spectra were internally normalized to C1*s* from adventitious carbon set to 285 eV. The S3*p*/S2*p* spectra in Figure 2 did not have an internal C1*s* scan, so they were

calibrated by setting the main $S2p_{3/2}$ from bulk CdS to the calibrated value of 161.5 eV. Background subtraction and fitting was performed using macros developed at SSRL using the data analysis software IGOR Pro. First, a Shirley-like background was subtracted from the spectra. In the peak fitting of the Shirley subtracted spectra, each doublet was represented by two Gaussians with a relative area ratio for the two spin orbit peaks ($2p_{1/2}$, $2p_{3/2}$) fixed at 1:2, and with a fixed spin-orbit split was also kept constant (1.2 eV for S2*p*).[4] These constraints led to quite stable fittings despite overlapping peaks. To evaluate the elemental concentration of each species, we evaluated the relative total areas of the fitted components, scaled by the relative sub-shell photoionization cross-section for each element according to Lindau and Yeah, for the two different excitation energies. The inelastic mean free path (IMFP) of the outgoing electrons gives a significant difference in probing depth between the 650 eV excitation (650-160 = 490 eV kinetic energy) and the 1080 eV excitation (1080-160 = 920 eV kinetic energy), which was used to evaluate the trend in the surface to bulk contributions. The IMFP for the photoelectrons are taken from the tabulated value of inorganic compounds (Table 5) in H. Shinotsuka, et al. *Surf. Interface Anal.* 2019, *51*, 427), using the value for CdS, which translates to an IMFP of 1.3 nm for S2*p* and Se3*p* at 650 eV and 2.1 nm at 1080 eV. Sulfur 2*p* XPS is very sensitive to the chemical state due to the wide redox range of sulfur and the difference in screening of the relatively shallow core level. Bulk sulfide (S^{2-}) in CdS can be observed at around 161.5 eV binding energy, and chemical shifts vary from smaller shifts associated with S^{1-} and S^0 species[5-10] all the way up to 8 eV shifts for highly oxidized sulphur (S^{4+}, S^{6+}), which has been observed on CdS QDs,[11] CdS buffer layers,[12] and CIGS surfaces.[13]

In Figure 5 (main article), we observe the high-resolution S2p spectra for fully "giant" nanocrystals: (a) gQD I and (b) gQD II. As opposed to the QDs with thinner CdS shell, these nanocrystals have a 15 ML CdS shell for which the Se3p photoelectrons do not penetrate the shell, and we observe only the S2p contribution to the region. In the case of gQD I, high-resolution S2p spectra fit well with two different spin–orbit split S-doublets. The spin-orbit doublet with binding energy of ~161.5 eV corresponds to inorganic sulfide atoms of the bulk crystal structure (S^{2-}).

In addition to the sulfide doublet, a second well-defined doublet is observed with a binding energy of ~163 eV which, based on the degree of chemical shift, can be associated with a S^{1-} oxidation state. We attribute the molecular origin of this S^{1-} chemical state to a surface-bound ligand, based on the chemical shift agreement with previous XPS-based observations of ligand-bound sulfur in CdS based absorbers,[13,6] QD nanocrystals,[11] and thiol-based SAMs (e.g., Ref. 8). The shifted component is too large to be attributed to a surface S atom of the CdS (surface core-level shifts of CdS is rather small ~0.4 eV).[4] The presence of thiol covalently bonded both to Cd and to carbon at the surface (Cd-S-C_x) can be further assessed when following growth of CdS via XPS on CdTe cores using thiol-based chemistry (e.g., Ref. 10). Since the CdTe does not have surface sulfur, we can more clearly see the single doublet from the bonded thiol in the initial phase (thiol-CdTe), and as the CdS shell is formed, we retort to a more broad doublet at lower energies associated with sulfide. Similar rather conclusive association of the S^{1-} feature to surface bound ligands can also be found in Ref. 11 (see, e.g., Figure 5 and Figure 12S and associated discussion) in which thiolate species are observed and can be seen to decompose upon X-ray radiation. The presence of a rather well-defined

doublet at 1-1.5 eV above bulk sulfide, and the broader bulk peak near sulfide observed for CdS terminated surfaces, matches very closely our XPS results for both gQD I and gQD II. For gQD II we observe a broader doublet (consisting of bulk and surface CdS and some off-lattice distortion contributions), whereas the sharper contribution from bonded thiol based sulfur is suppressed. Due to overlapping features, we cannot completely rule out minor contributions from S^{1-} to the gQD II spectrum, nor from S^0 at slightly higher energies. However, these data are consistent with the absence of thiol in the case of gQD II.

Powder X-ray diffraction (XRD) analysis: XRD data were collected using a Scintag powder XRD powder X-ray diffractometer in Bragg-Brentano geometry (Figure S4), or on a Rigaku SmartLab II diffractometer in parallel beam geometry (Figure S8). To optimize XRD pattern quality and remove artifacts arising from excess ligands, the synthesized gQDs were cleaned by precipitation with ethanol followed by redissolution in hexane. The procedure was repeated 2-3 times, before being drop casted and vacuum dried SiO_2 substrate. However, care was taken to avoid "over-cleaning" of dots by ethanol precipitation (>3 times), in which case the relative intensities of the peaks were found to be altered, due to preferential packing/clustering of gQDs along a preferred direction due to lack of ligands.

Powder XRD analysis provided insight into the crystallographic nature of gQDs. Figure S4 shows the XRD patterns for gQD I and II. Starting from wurtzite (*W*) CdSe cores, both the gQDs exhibit *W* type growth pattern, having the characteristic <100>, <002>, <101>, <102>, <110>, <103> and <112> peak reflections typical for *W*-CdS (see

insets). However, the relative intensities of the peaks deviate from the bulk pattern. Especially, the <102> and <103> peak reflections in the gQDs (markedly more prominent in the case of gQD II, Figure S4b) are observed to be attenuated, which is indicative of the existence of zinc blende (*ZB*) stacking faults along the <002> direction as reported earlier.[13] Rietveld refinement analysis was performed on all XRD patterns using either Jade 9.0 software (WPF + Rietveld; Figure S4) or PDXL package v2.6 (Figure S8; Rwp = 4.2-4.5% and S = 1.66-1.67). For gQD I the analysis yielded a Greenockite CdS (*W*) phase [PDF#01-077-2306] contribution of ~95% and Hawleyite CdS (*ZB*) phase [PDF#01-080-0019] contribution of ~5%, with an R value 7.81 %. While for gQD II, the refinement analysis yielded a Greenockite CdS (*W*, hexagonal, P63mc) phase [PDF#01-077-2306] contribution of ~65% and Hawleyite CdS (*ZB*, cubic, F-43m) phase [PDF#01-080-0019] contribution of ~35%, with an R value 4.8%. To confirm the data consistency between the two different diffractometers, the gQD I sample was reanalyzed in the parallel beam geometry on the Rigaku diffractometer, and the data refinement in PDXL package produced the same results as those obtained with Rietveld refinement using Jade (98% *W* compared to 95%).

NMR analysis: Prior to analysis, gQDs were washed via 5-6 cycles of precipitation (anhydrous ethanol)/resuspension (hexane) in an inert atmosphere glovebox, followed by vacuum drying for ~1 h to remove volatile impurities. Dried gQDs were dispersed in anhydrous $CDCl_3$ (0.8 mL; ~50 nM), and transferred to Wilmad-Labglass 5 mm NMR sample tubes and sealed. NMR spectra were acquired using a Bruker Avance 500 at a magnetic field strength of 11.7 T (^{1}H frequency of 500.13 MHz) with a tunable 5 mm broadband probe with ^{2}H lock at 76.773 MHz. All spectra are referenced internally to

$CDCl_3$ (7.26 ppm). ^1H NMR spectra were obtained for pure ligands, OT, OAc, Olam (**Figure S5**) and those for gQD I, gQD I with added octanethiol, and gQD II, (**Figure S6a-c**). Peaks identified in pure-ligand spectra were used in conjunction with literature precedent to elucidate peaks obtained from gQD suspensions. Ligand-only spectra were obtained using identical experimental conditions as used for the gQD sample analyses.

For clarity, only the relevant sections of the NMR spectra obtained for the gQD samples are depicted in Fig. S6, i.e., the $CDCl_3$ peak (δ = 7.26 ppm) and methylene (δ = 1.258 ppm) and methyl (δ = 0.882 ppm) protons of alkyl groups of the ligands are not shown. Broad signals indicate that ligands are bound to (or in rapid dynamic exchange with) the nanocrystal surface, which can also result in peak shifting.[14-18]

In addition, a number of peaks (discussed below) indicate the likely presence of oleylamine-oleic acid condensation products along with the pure moieties (e.g., oleate, which is present in excess), an observation reported in recent literature.[19-24] The formation of amides as the product of acid-base condensation or salt (ion pair) formation is expected in QD reactions that are conducted at elevated temperature. In the following discussion, R, R' refer to the alkyl part of oleic acid R = $C_{17}H_{33}$ and oleylamine (R' = $C_{18}H_{35}$), respectively. Amide moeity refers to both oleamide ($RCON\underline{H_2}$) or dioleamide ($RCON\underline{H}R'$) species. Notably, formation of acid-base adducts (salt formation, i.e., $[R'NH_3^+][RCOO^-]$) results in a downfield and upfield shift of the α H peaks of oleylamine and oleic acid, respectively, compared to peak positions of the ligand-only ^1H NMR spectra. Specifically, protonation of the amine group leads to deshielding of α H peaks, resulting in the downfield shift, while corresponding acid group deprotonation leads to increased shielding of α H peaks, and thus associated upfield shift in the case of oleic acid. Again, following the

formation of amide, which might exist as the amide-iminol tautomer (keto-enol tautomerism),[19] identical partial charge separation over the 'N' atom and carboxylic 'C' atom of amide leads to similar shifts in the ^{1}H NMR spectra.

Analysis of gQD I ^{1}H NMR (Figure S6a; Table S2): Peak **A**, δ = 5.6 ppm, is ascribed to the amidic proton.[19] Peak **B**, δ = 5.346 ppm, is ascribed to vicinal protons of the 9,10 unsaturation [C(9)H=C(10)H] in oleic acid/oleylamine or their condensation products. The trio of peaks (**C**) from δ = 4.966-4.648 ppm results from the terminal double bond of the 90% technical grade ODE employed for syntheses and an impurity found in it.[14] Peak **D**, δ = 3.8 ppm, is assigned to residual ethanol employed for QD cleanup, downfield shifted from characteristic peak position at δ = 3.72 ppm. Peak **E**, δ = 3.391 ppm, is ascribed to protons on carbon α to the nitrogen atom of formed amide [(R'''-CH2-NH-C(=O)R, R'', alkyl = R'''- CH_2], shifted downfield due to charge separation resulting from keto–enol tatutomerism. The peak **F**, δ = 2.769 ppm, appears in the region where one would expect the α-CH_2 protons of surface-bound octanethiol to appear,[18] shifted downfield from the free ligand signal at δ = 2.51 ppm (Figure S5, OT NMR). The observation of this signal was inconsistent across a number of samples prepared, possibly indicating that this signal arises from an impurity or that the observation of the weak signal for surface-bound thiol(ate) is concentration dependent. Signal **G**, δ = 2.351 ppm, is consistent with the presence of free oleic acid in solution. Signal **H**, δ = 2.221 ppm appears as a sharp triplet, consistent with the α-CH_2 group of an unbound derivative of OAc/Olam, possibly in the form of a condensation product or oleate salt. Peak **I**, δ = 2.034-2.005 ppm, is ascribed to protons on carbon atoms allylic to the 9,10 unsaturation of oleic acid, oleylamine or amide moieties, and also 1,2 unsaturation of ODE.

The addition of a small amount (2 μL) of 1-octanethiol to the gQD I suspension results in several changes observed by NMR spectroscopy (Figure S6b; Table S3). A broad signal **F'** (δ = 2.769 ppm) appears in the region where the signal **F** had been observed prior to OT addition. The signal is broad, indicative of a gQD surface-bound thiol species. A new peak **X** appears at δ = 2.696-2.652 ppm, which is assigned to di-n-octyl disulfide species formed due to partial oxidation of excess added thiol species. The sharpness of this peak indicates that this species is not surface bound. Likewise, emergence of an additional sharp peak **Y**, δ = 2.523 ppm, is assigned to free octanethiol. The peak **G'** (δ = 2.350 ppm), previously identified as free oleic acid (Figure S6a and Table S2, peak **G**), is observed to become more intense. This likely results from the displacement of surface bound oleic acid/oleate by the newly introduced thiol species.

Analysis of gQD II ^{1}H NMR (Figure S6c; Table S4): Using the same conditions, ^{1}H NMR was obtained for gQD II (Figure S6c). The intense peak **P**, δ = 5.336 ppm, is ascribed to vicinal protons of the 9,10 (<u>CH</u>=<u>CH</u>) unsaturation in oleic acid/oleylamine or their condensation products, (gQD I peak **B**). The two weak peaks, **Q**, δ = 4.725 and 4.647 ppm, result from the terminal double bond of the residual ODE (gQD I **C** peaks, not including impurity at 4.966 ppm). Peak **R**, δ = 3.7221 ppm, is assigned to residual ethanol impurity (gQD I peak **D**). Peak **S**, δ = 3.6435 ppm, is ascribed to protons on carbon α to nitrogen atom of formed acid- base adduct (ion pair [R'''- C$\underline{H_2}$-NH_3^+][$RCOO^-$], R'=R'''- CH_2) bonded with QD surface. Peak **T** corresponds to peak **G** in gQD I, peak **U** to peak **H**, and peak **V** to **I** (see discussion above).

Single-QD photoluminescence microscopy and spectroscopy: Optical microscopy under stress conditions of heat and high photon flux—Suspensions of gQDs with concentrations on the order of 1 nM were prepared by dilution in hexanes. A small amount of the ultra-dilute suspension was dropcast onto a glass coverslip. Substrates were then loaded into a cryostat (Oxford Microstat HiRes) for high-temperature optical experiments. A widefield continuous-wave (CW) 405 nm source was used for constant illumination. The spot size of the beam was expanded to ∼50 μm in diameter using an f = 25 mm lens, and the average pump fluence was set to either ∼1 W/mm^2 or 15 W/mm^2. Room-temperature blinking and photobleaching experiments were performed under the pump fluence of 1 W/mm^2. For temperature-dependent photoluminescence experiments, the sample temperature was cycled from room temperature to ~100 °C and back to room temperature over ~2.5 h under the ultra-high pump fluence of 15 W/mm^2. For long-term photobleaching experiments, gQDs were subjected to a constant photon flux of 1 W/mm^2 and temperature of ~100 °C. In this case, the total illumination time was up to 11 h. The average intensity and number of single gQDs at the end of each time interval were extracted using the ImageJ image processing program.

Fluorescence-lifetime intensity distribution (FLID) plots and determination of QD charging[24-26]—QDs dropcast on a glass coverslip were imaged by raster scanning with a 405 nm pulsed excitation source (PicoQuant PDL; 30 ps pulse width, 2.5-10 MHz repetition rate), with the repetition rate chosen to ensure that the PL lifetime trace decayed fully before the arrival of subsequent pulses. The setup was described in Ref. 25. The number of excitations per pulse ⟨N⟩ was less than 0.2, and emitted photons were collected onto two avalanche photodiodes (APDs; SPCM-AQRH-14, PerkinElmer) in a

Hanbury–Brown Twiss interferometric configuration. Homebuilt software was used to perform all analysis of photoluminescence data and generate FLIDs. Representative FLIDs characteristic of an uncharged (neutral) and a charged gQD, respectively, are shown along with their accompanying structural and chemical imaging, intensity vs. time trace, and fluorescence decay curve (Figure S9). An uncharged QD has a FLID with intensity centered on a longer fluorescence decay lifetime and higher fluorescence intensity, with a tail extending toward shorter fluorescence decay lifetime and lower fluorescence intensity. In contrast, a charged QD has a FLID with intensity centered on a shorter fluorescence decay lifetime and lower fluorescence intensity, with a tail extending toward longer fluorescence decay lifetime and higher fluorescence intensity. QDs were classified as charged or uncharged based on which of the two states showed a higher probability during the interrogation time (~300 s). Because the lifetime and intensity of the QD photoluminescence vary over time, both are calculated for each time bin (every 100 ms); plotting the lifetime *vs* intensity for each bin provides the 2D heatmap histograms shown in Figure S9c,g. This representation facilitates visualization of exciton dynamics over long time scales.

Single-dot FWHM were measured using the above setup with widefield continuous wave 405 nm excitation from a PicoQuant PDL 405 laser. The emission from a single QD was then focused onto a liquid nitrogen cooled CCD camera (Princeton Instruments) with integrated spectrophotometer. This process was repeated for multiple individual QDs to yield the results shown in Figure 7a.

Determining second-order fluorescence intensity correlation ($g^{(2)}$)—The Hanbury Brown Twiss apparatus was also used to determine $g^{(2)}$ plots using the analysis described previously.[26-27]

Statistical Analysis: Data is presented where applicable as mean ± standard deviation. ImageJ was used to analyze particle size. Sample size is provided in figure legends, within figures and/or tabulated form. With the exception of Figure S3, right (as indicated), TEM images were not processed/filtered.

Supporting Figures

a

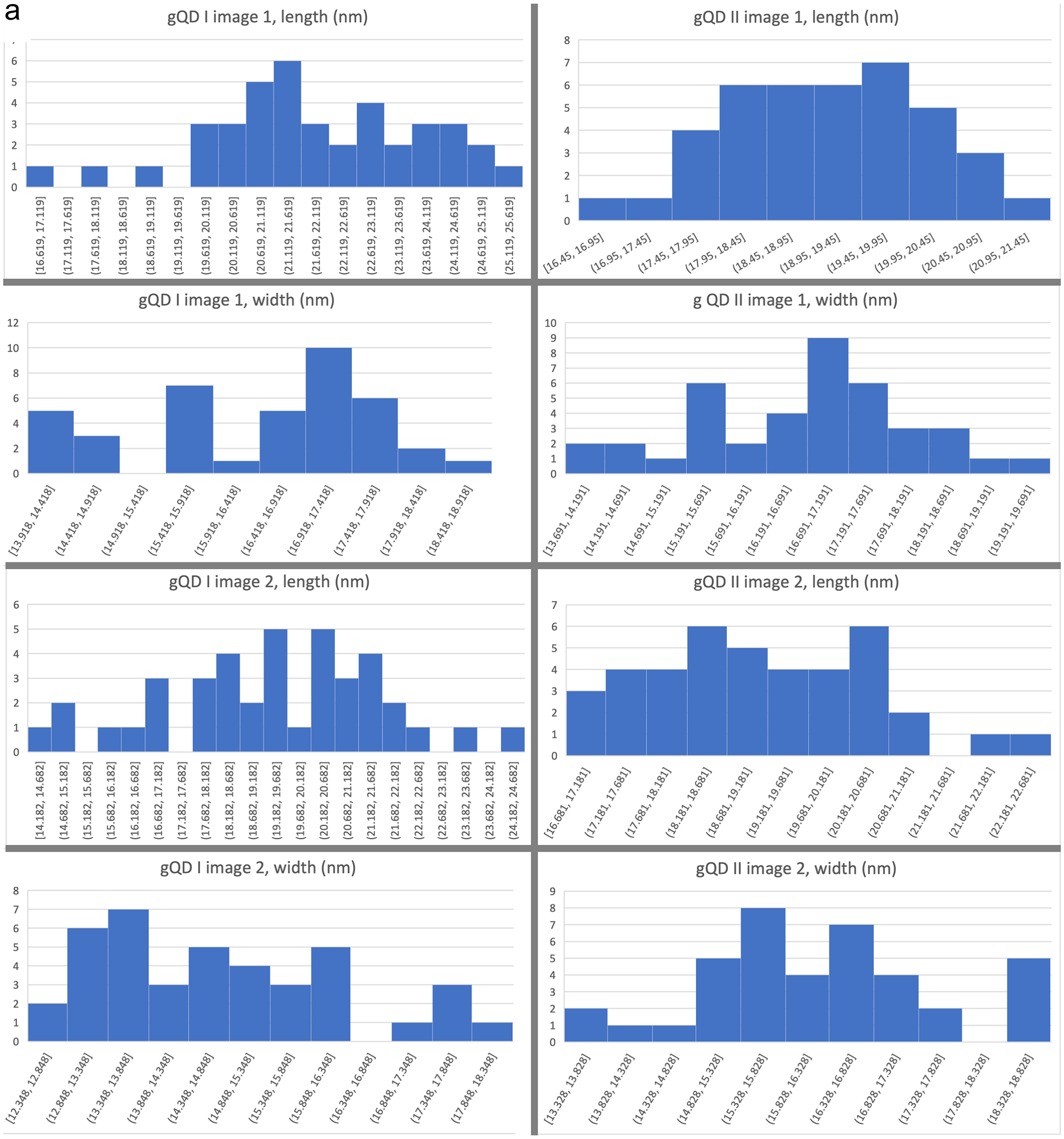

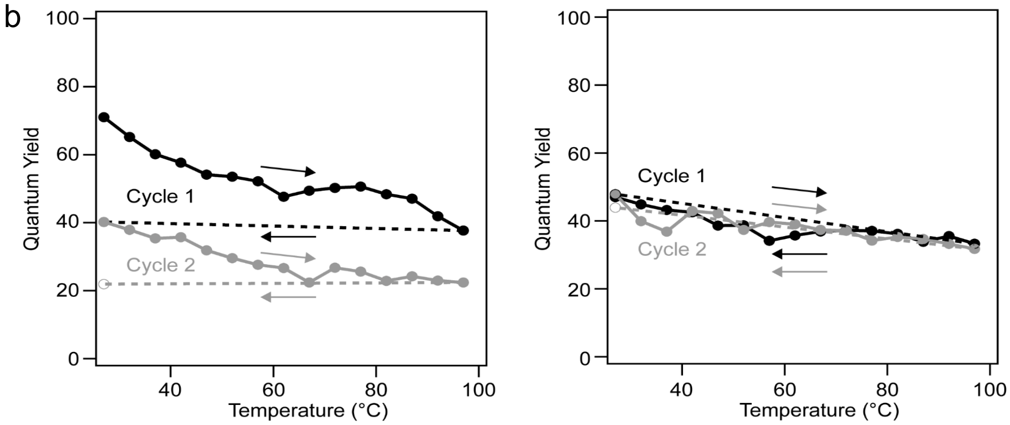

Figure S1. (a) Size histograms for gQD I and gQD II obtained in each case from analysis of length and width of 80 nanocrystals (40 from each of 2 images for (b) Photoluminescence intensity in absolute quantum yield as a function of temperature for gQD I (left) and gQD II (right) under high photon flux (1 W/mm^2). Clear differences in the extent of photobleaching and nature of recovery upon cooling are observed for two heating-cooling cycles (~2.5 h of illumination per cycle). gQD I both loses intensity and does not recover in each cycle, while gQD II loses a smaller percentage of its starting intensity and recovers in both cycles.

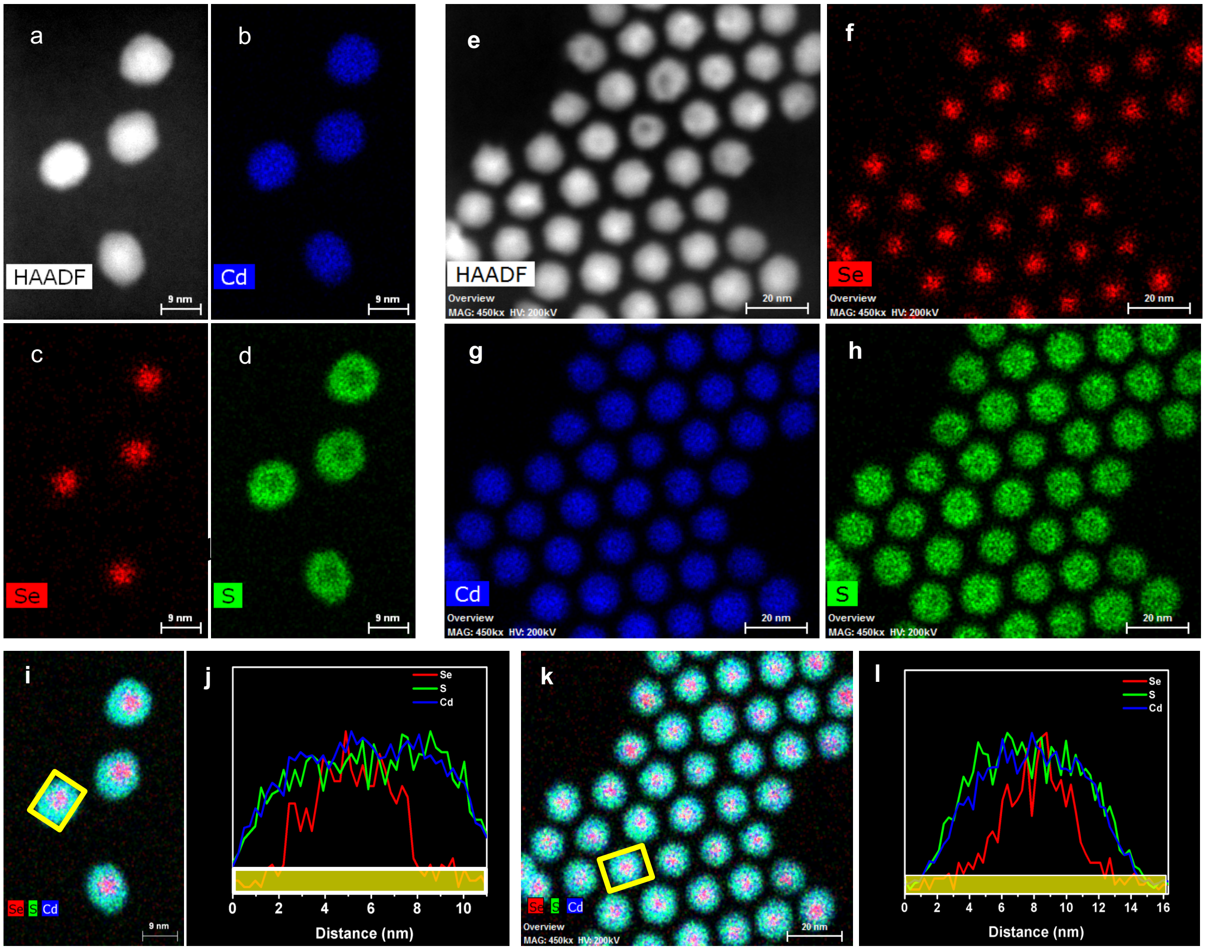

Figure S2. STEM-EDS images of core/shell nanocrystals possessing ~9 ML of CdS shell. (a) HAADF-STEM image of 4 NCs prepared by the continuous injection method, akin to gQD I. (b)-(d) Elemental mapping showing Cd, Se, S, respectively. (e) HAADF-STEM image of many NCs prepared by the SILAR growth method, akin to gQD II. (f)-(h) Elemental mapping showing Cd, Se, S, respectively. (i) Composite elemental maps for NCs in (a). (j) EDS line-scan analysis of the NC boxed in (i). (k) Composite elemental maps for NCs in (e). (l) EDS line-scan analysis of the NC boxed in (k).

Table S1. Summary of average Se penetration into the CdS shell obtained for each gQD type by EDS analysis in each case of ~15-30 nanocrystals.

**QD type**	**CdSe core diameter**	**# Single QDs analyzed**	**CdSe/ CdS diameter**	**# Single QDs analyzed**	**Average Se signal (nm)**	**Radial extension of Se into shell**
gQD 1	5.2 nm	30	11.3 ± 0.1 nm	4	6.0 ± 0.4	**0.4 ± 0.20 nm (main text Figure 2)**
gQD 1 (different batch; used for anneal study: see bottom row, and for XPS)	4.7 nm	15	10.3 ± 1.1 nm	22	5.6 ± 0.3	0.45 ± 0.15 nm
gQD 2	4.8 nm	30	12.8 ± 1.0 nm	40	8.5 ± 0.4	**1.85 ± 0.20 (main text Figure 2)**
gQD 2 (different batch; used for XPS)	4.9 nm	20	11.2 ± 2.4 nm	78	8.75 ± 0.60	1.92 ± 0.30
gQD 1 post-38 h anneal	4.7 nm	15	N/A	N/A	7.24 ± 0.44	1.27 ± 0.22

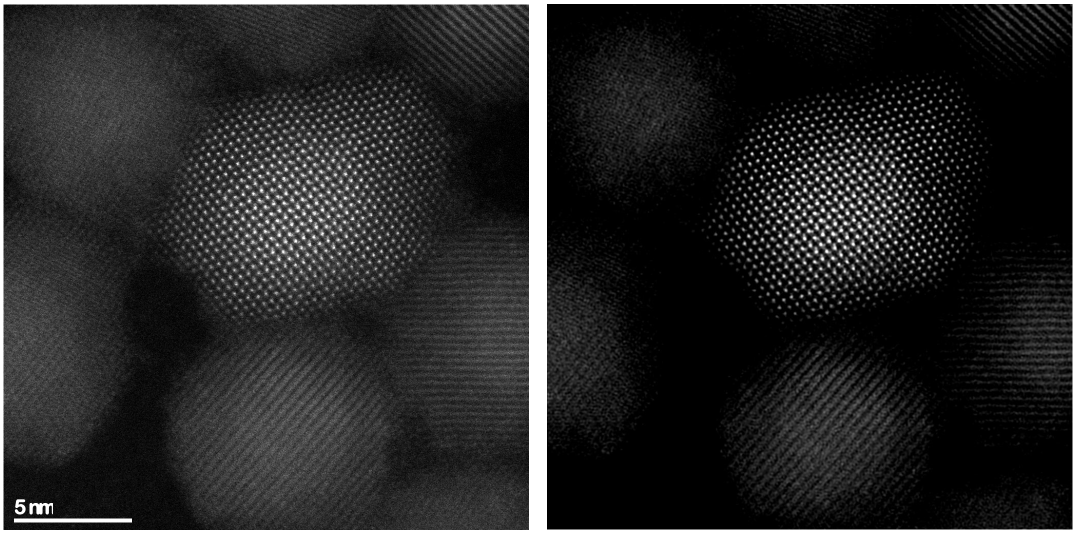

Figure S3. Aberration-corrected HAADF-STEM images of CdSe/CdS QD comprising a 6 ML shell synthesized by the continuous-injection method: original image (left) and filtered image to remove some background (right).

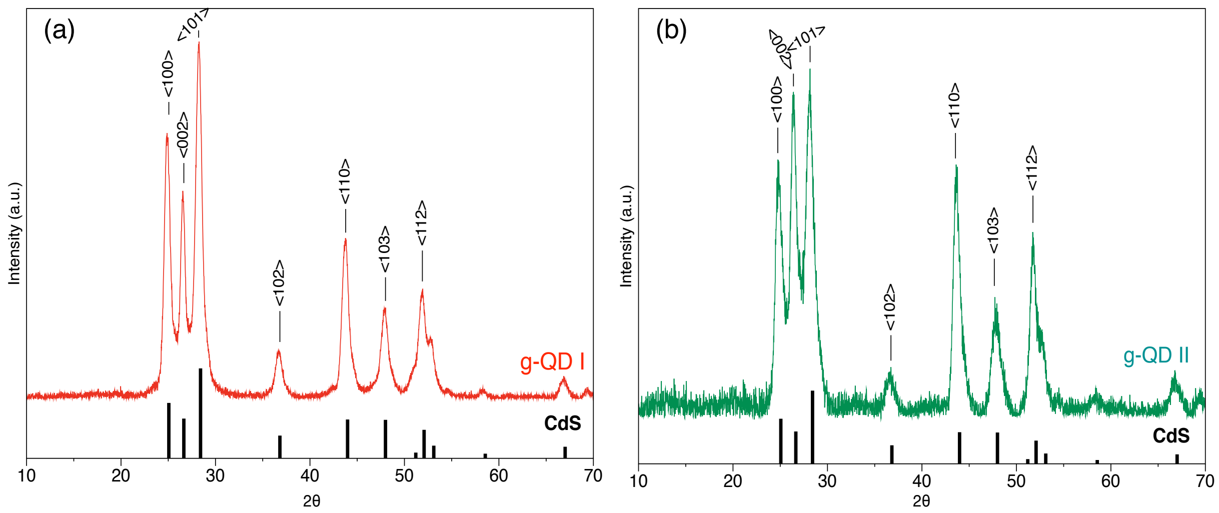

Figure S4: Powder XRD patterns for gQD I (a) and II (b) shown at 15 MLs shell thickness. The XRD pattern for bulk CdS (*W*) (black sticks) standard is provided for reference.

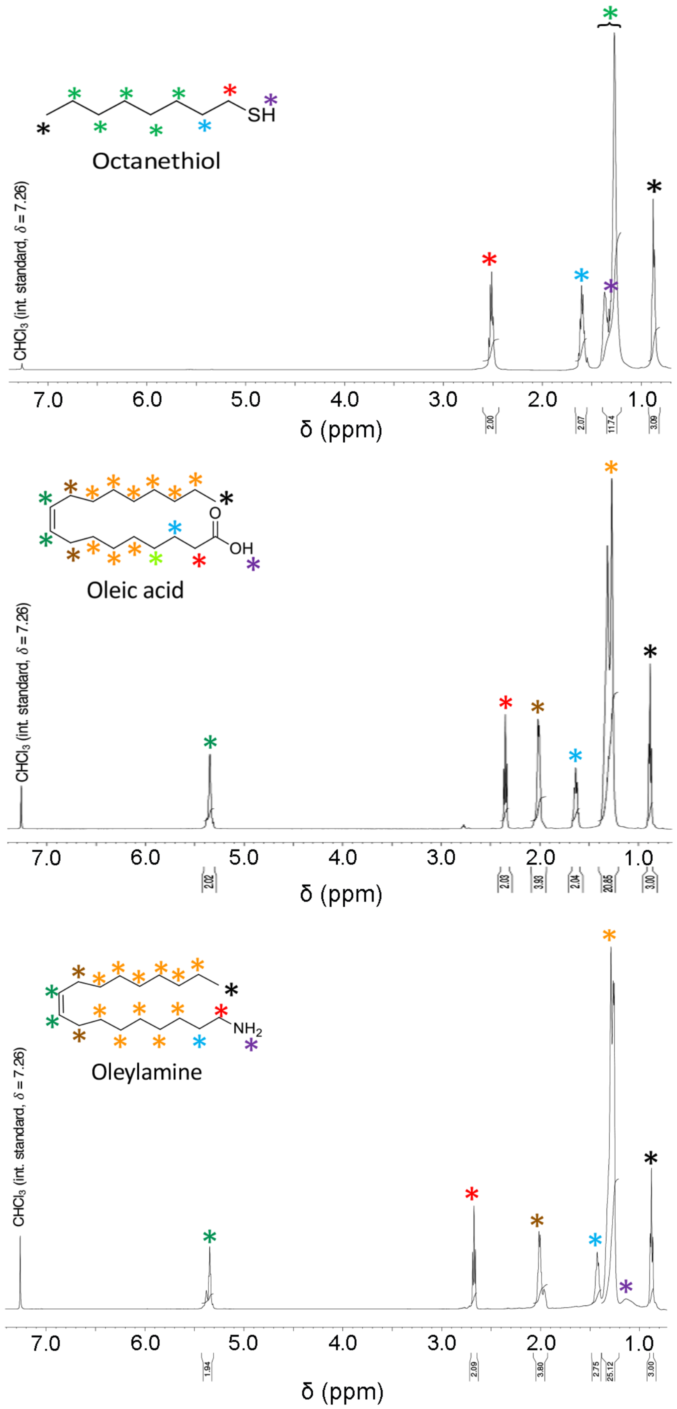

Figure S5. ^{1}H NMR spectra for octanethiol (OT), oleid acid (OAc) and oleylamine (Olam) obtained in $CDCl_3$.

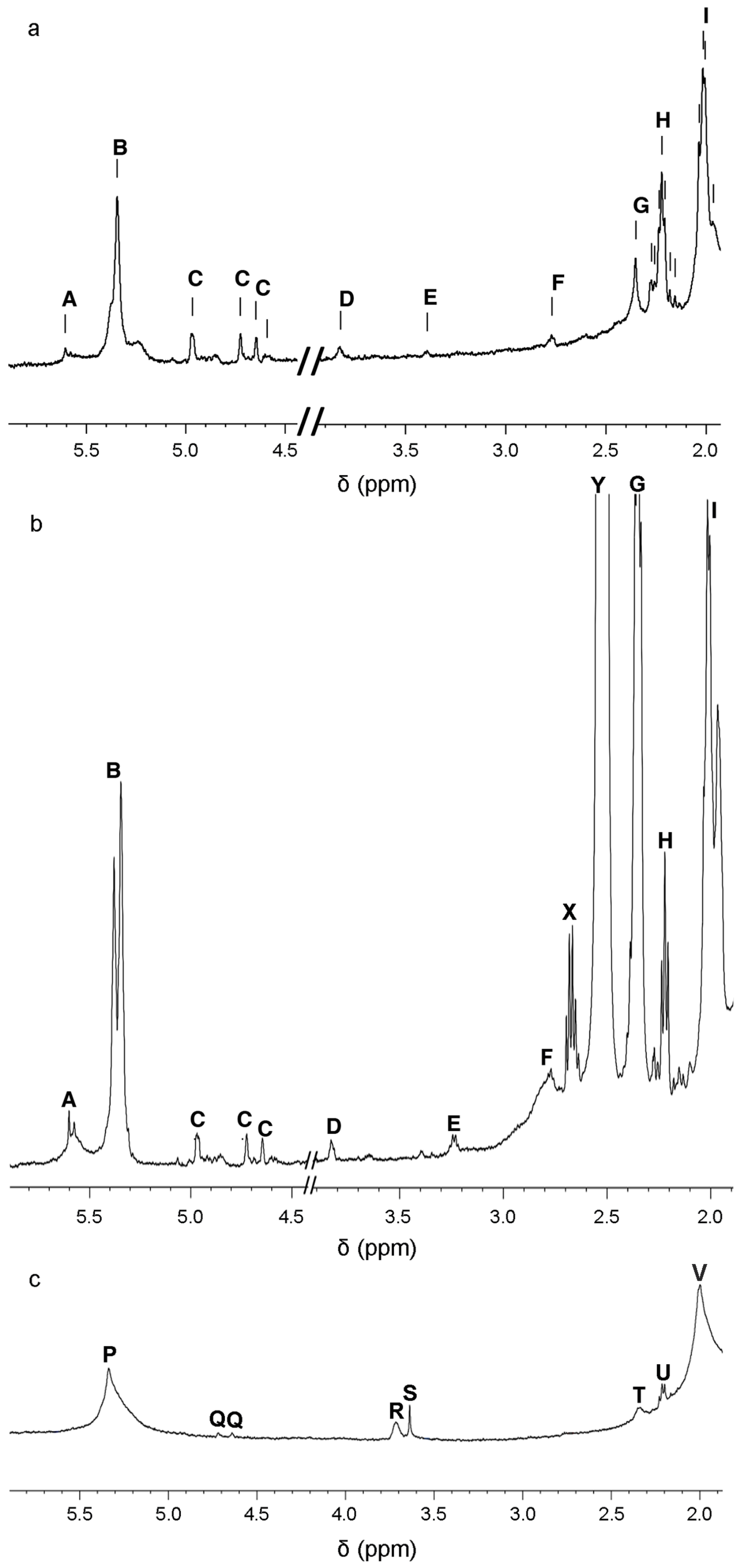

Figure S6. ^{1}H NMR spectra for (a) gQD I, (b) gQD I + octanethiol, (c) gQD II in $CDCl_3$.

Table S2. Summary of ^{1}H NMR peaks observed in gQD I sample.

Sample	Peak(s) denoted by	Observed chemical shift δ (ppm)	Remarks (R, R', R" = Alkyl group)
gQD I	A	5.6	OAc/Olam condensation product or amide hydrogen R (C= O) NHR', R (C= O) NH_2
	B	5.346	Vinylic H of 9,10 unsaturation of OAc, Olam or oleamide/dioleamide R-CH_2-CH=CH-CH_2-R"
	C	4.966 - 4.648	Vinylic Hydrogens attached to terminal 1,2 unsaturation of ODE and an impurity occurring in it R-CH=CH_2
	D	3.8	Residual ethanol from the gQD purification protocol, downshifted (CH_3-CH_2-OH)
	E	3.391	Protons on C atoms α to amide N, in the formed amide, shifted downfield
	F	2.769	Signal in the region where protons on C atoms α to thiol group, RCH_2-SH, are expected to appear
	G	2.351	Protons on C atoms α to carboxylate group of OAc (RCH_2COOH)
	H	2.235 - 2.205	Protons on C atoms α to carboxylate group of oleate (RCH_2COO^-) or a derivative of Oac/Olam (condensation product or salt)
	I	2.034 -2.005	Protons on carbon atoms allylic to the (a) 9,10 unsaturation of OAc, Olam or amide moieties and (b) 1,2 unsaturation of ODE RCH_2CH=$CHCH_2R"$

Table S3. Summary of ^{1}H NMR peaks observed in gQD I + thiol

Sample	Peak(s) denoted by	Observed chemical shift δ (ppm)	Remarks (R, R', R" = Alkyl group)
gQD I + OctSH	A'	5.6	Peak corresponding to peak A
	B'	5.346	Peak corresponding to peak B
	C'	4.966 - 4.648	Peak corresponding to peak C
	D'	3.8	Peak corresponding to peak D
	E'	3.391	Peak corresponding to peak E
	F'	2.769	Peak corresponding to peak F, more intense and broad
	X	2.696 – 2.652	Peak assigned to Di-N-octyl disulfide species, unbound, sharp
	Y	2.544-2.5	Peak assigned to free unbound OT
	G'	2.351	Peak corresponding to unbound OAc peak G, intensified due to liberation of bound oleate
	H'	2.235 - 2.205	Peak corresponding to peak H
	I'	2.034 - 2.005	Peak corresponding to peak I

Table S4. Summary of ^{1}H NMR peaks observed in gQD II

Sample	Peak(s) denoted by	Observed chemical shift δ (ppm)	Remarks (R,R',R" = Alkyl groups)
gQD II	P	5.3426	Vinylic hydrogen atoms of 9,10 unsaturation of OAc, OlAm or their condensation product R-CH_2-C(9)H=C(10)H-CH_2-R"
	Q	4.725 - 4.647	Vinylic hydrogens attached to terminal 1,2 unsaturation of 90% pure commercial ODE and an impurity occurring in it R-C(1)H=C(2)H_2
	R	3.7221	Residual ethanol from the purification protocol
	S	3.6435	Protons on C atoms α to protonated N, in the acid base adduct, shifted downfield [R''-CH_2-NH_3^+][$RCOO^-$]
	T	2.3439	Protons on C atoms α to carboxylate group of OAc (RCH_2COOH)
	U	2.2339 - 2.2043	Protons on C atoms α to carboxylate group of oleate (RCH_2COO^-) or a derivative of Oac/Olam (condensation product or salt)
	V	2.0042	Protons on carbon atoms allylic to the 9,10 unsaturation of OAc, Olam, acid-base adduct RCH_2CH=$CHCH_2R$" and also 1,2 unsaturation of ODE (RCH_2CH = CH_2)

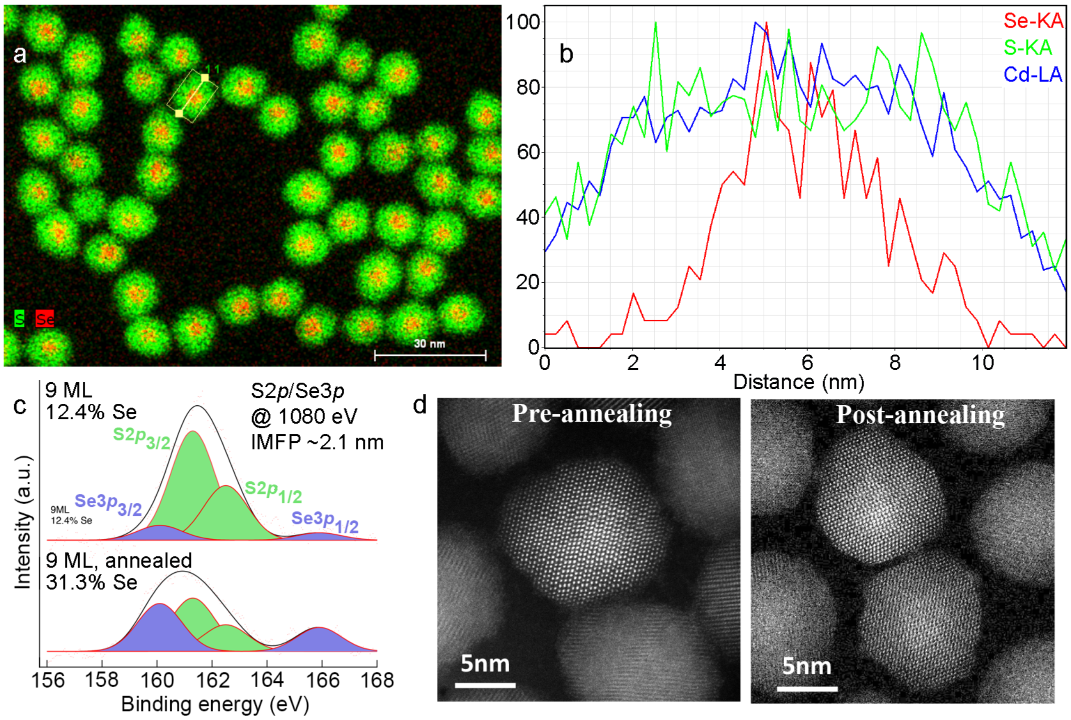

Figure S7. Effects of annealing on Se-S mixing: (a) Composite STEM-EDS map of Se and S for post-anneal ~9 ML CdSe/CdSe QDs prepared using continuos injection. (b) EDS line-scan analysis of the NC in (a). (c) XPS spectra for continuous-injection CdSe/CdS core/shell QDs (9 ML) pre- and post-anneal; Se signal increases with annealing; doublet peaks for S 2*p* and Se 3*p* orbitals obtained using a photon energy of 1080 eV. (d) HAADF-STEM images of representative QDs in (a)-(c) pre- and post-anneal.

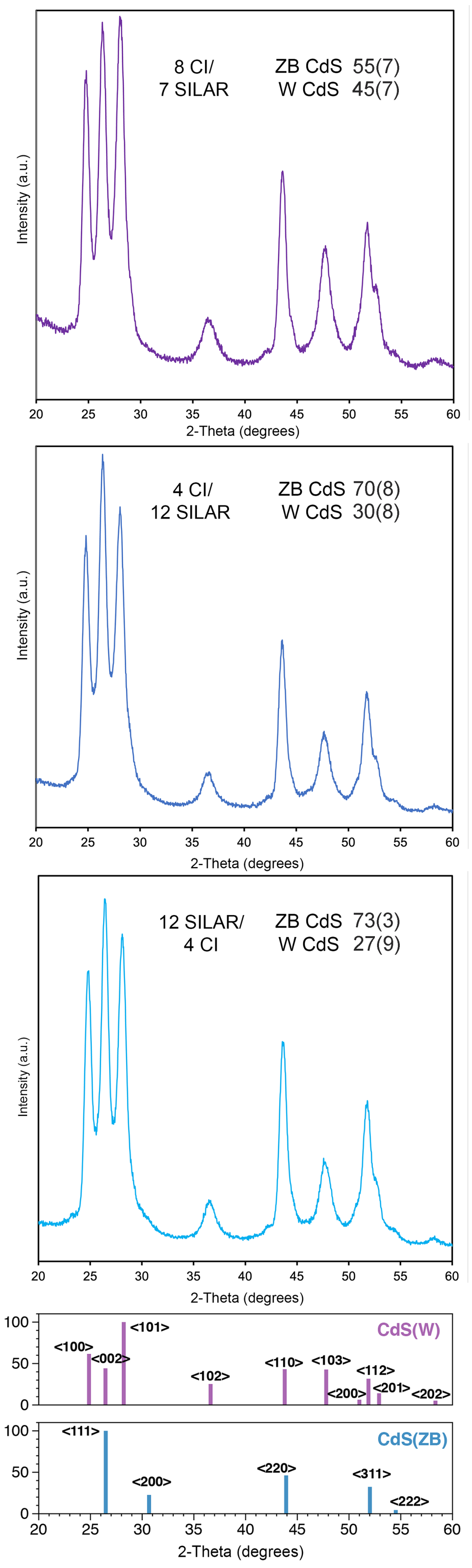

Figure S8. Powder XRD patterns for mixed-synthesis gQDs. Reference patterns are shown for bulk CdS wurtzite and zinc blende structures.

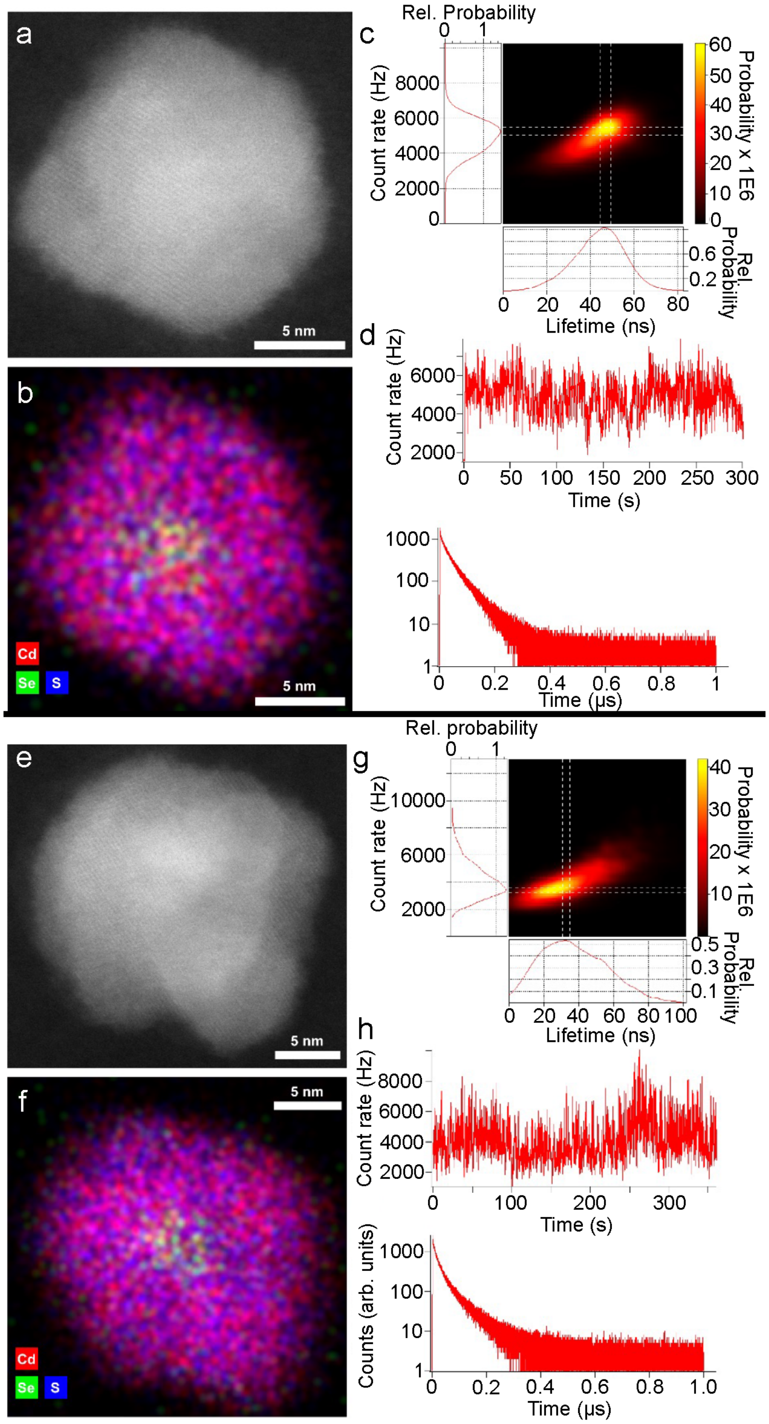

Figure S9. (a)-(d) and (e)-(h) are data obtained from a representative uncharged and charge gQD, respectively. (a),(e) HAADF-STEM images; (b),(f) STEM-EDS images; (c),(g) FLID plots; (d),(h) photoluminescence time traces and photoluminescence decay lifetimes.

References:

1. L. Carbone, C. Nobile, M. De Giorgi, F. D. Sala, G. Morello, P. Pompa, M. Hytch, E. Snoeck, A. Fiore, I. R. Franchini, M. Nadasan, A. F. Silvestre, L. Chiodo, S. Kudera, R. Cingolani, R. Krahne, L. Manna, *Nano Lett.* **2007**, *7*, 2942.
2. O. Chen, J. Zhao, V. P. Chauhan, J. Cui, C. Wong, D. K. Harris, H. Wei, H.-S. Han, D. Fukumura, R. K. Jain, M. G. Bawendi, *Nature Mater.* **2013**, *12*, 445.
3. Y. Ghosh, B. D. Mangum, J. L. Casson, D. J. Williams, H. Htoon, J. A. Hollingsworth, *J. Am. Chem. Soc.* **2012**, *134*, 9634.S. Wiklund, K. O. Magnusson, S. A. Flodström, *Surface Science* **1990**, *238*, 187.
4. V. Dzhagan, O. Selyshchev, S. Kondratenko, N. Mazur, Y. Havryliuk, O. Raievska, O. Stroyuk, D. R. T. Zahn, *Electronic Materials* **2022**, *3*, 136.
5. O. Selyshchev, Y. Havryliuk, M. Ya. Valakh, V. O. Yukhymchuk, O. Raievska, O. L. Stroyuk, V. Dzhagan, D. R. T. Zahn, *ACS Appl. Nano Mater.* **2020**, *3*, 5706.
6. B. R. C. Vale, R. S. Mourão, J. Bettini, J. C. L. Sousa, J. L. Ferrari, P. Reiss, D. Aldakov, M. A. Schiavon, *Sci Rep* **2019**, *9*, 8332.
7. D. G. Castner, K. Hinds, D. W. Grainger, *Langmuir* **1996**, *12*, 5083.
8. S. Wiklund, K. O. Magnusson, S. A. Flodström, *Surface Science* **1990**, *238*, 187.
9. H. Peng, L. Zhang, C. Soeller, J. Travas-Sejdic, *J. Lumin.* **2007**, *127*, 721.
10. B. R. C. Vale, R. S. Mourão, J. Bettini, J. C. L. Sousa, J. L. Ferrari, P. Reiss, D. Aldakov, M. A. Schiavon, *Sci. Rep.* **2019**, *9*, 8332.
11. D. A. Duncan, J. M. Kephart, K. Horsley, M. Blum, M. Mezher, L. Weinhardt, M. Häming, R. G. Wilks, T. Hofmann, W. Yang, M. Bär, W. S. Sampath, C. Heske, *ACS Appl. Mater. Interfaces* **2015**, *7*, 16382.
12. V. Dzhagan, O. Selyshchev, S. Kondratenko, N. Mazur, Y. Havryliuk, O. Raievska, O. Stroyuk, D. R. T. Zahn, *Electronic Materials* **2022**, *3*, 136.
13. Y. Gao, X. Peng, *J. Am. Chem. Soc.* **2014**, *136*, 6724.
14. R. Gomes, A. Hassinen, A. Szczygiel, Q. Zhao, A. Vantomme, J. C. Martins, Z. Hens, *J. Phys. Chem. Lett.* **2011**, *2*, 145.
15. Z. Hens, J. C. Martins, *Chem. Mater.* **2013**, *25*, 1211.
16. Y. Shen, M. Y. Gee, R. Tan, P. J. Pellechia, A. B. Greytak, *Chem. Mater.* **2013**, *25*, 2838.
17. Y. Shen, A. Roberge, R. Tan, M. Y. Gee, D. C. Gary, Y. Huang, D. A. Blom, B. C. Benicewicz, B. M. Cossairt, A. B. Greytak, *Chem. Sci.* **2016**, *7*, 5671.
18. M. R. McPhail, E. A. Weiss, *Chem. Mater.* **2014**, *26*, 3377.
19. M. B. Mohamed, K. M. AbouZeid, V. Abdelsayed, A. A. Aljarash, M. S. El-Shall, *ACS Nano* **2010**, *4*, 2766.
20. W. Niu, S. Wu, S. Zhang, *J. Mater. Chem.* **2010**, *20*, 9113.
21. W. Niu, S. Wu, S. Zhang, *J. Mater. Chem.* **2011**, *21*, 10894.
22. P. E. Chen, N. C. Anderson, Z. M. Norman, J. S. Owen, *J. Am. Chem. Soc.* **2017**, *139*, 3227.
23. R. A. Harris, P. M. Shumbula, H. Van Der Walt, *Langmuir* **2015**, *31*, 3934.
24. S. Mourdikoudis, M. Menelaou, N. Fiuza-Maneiro, G. Zheng, S. Wei, J. Pérez-Juste, L. Polavarapu, Z. Sofer, *Nanoscale Horiz.* **2022**, *7*, 941.

25. C. Galland, Y. Ghosh, A. Steinbrück, M. Sykora, J. A. Hollingsworth, V. I. Klimov, H. Htoon, *Nature* **2011**, *479*, 203.
26. C. Galland, Y. Ghosh, A. Steinbrück, J. A. Hollingsworth, H. Htoon, V. I. Klimov, *Nat Commun* **2012**, *3*, 908.
27. N. J. Orfield, J. R. McBride, F. Wang, M. R. Buck, J. D. Keene, K. R. Reid, H. Htoon, J. A. Hollingsworth, S. J. Rosenthal, *ACS Nano* **2016**, *10*, 1960.
28. Y.-S. Park, A. V. Malko, J. Vela, Y. Chen, Y. Ghosh, F. García-Santamaría, J. A. Hollingsworth, V. I. Klimov, H. Htoon, *Phys. Rev. Lett.* **2011**, *106*, 187401.
29.
